# How to evaluate long-term care insurance policy based on policy tools and PMC index model: evidence from pilot cities in China

**DOI:** 10.3389/fpubh.2025.1661785

**Published:** 2025-10-06

**Authors:** An-Qi Wang, Jiang-Na Wang, Yi-Chun Gu, Ni Yuan, Yi-Han Wu, Sheng-Nan Duan, Ji Luo, Chong-Jin An

**Affiliations:** ^1^School of Public Health, Dalian Medical University, Dalian, Liaoning, China; ^2^School of Management and Economics, Jiangxi University of Chinese Medicine, Nanchang, Jiangxi, China; ^3^Shanghai Health Development Research Center (Shanghai Medical Information Center), Shanghai, China; ^4^Medical Insurance Department, The First Hospital of China Medical University, Shenyang, Liaoning, China

**Keywords:** long-term care insurance, policy evaluation, policy tools, PMC index model, pilot cities, China

## Abstract

**Objective:**

To address the risk of disability arising from population aging, the long-term care insurance (LTCI) policy in China has been progressively piloted and expanded. This study aims to examine the tool combination and strengths and weaknesses of the LTCI policy through a textual quantitative analysis of policies issued in 29 pilot cities, thereby providing a reference for refining the policy framework.

**Methods:**

Seventy-nine LTCI policies were analyzed based on the two-dimensional analytical framework regarding policy tools and policy ratings. Policy texts were coded and analyzed according to the connotative elements of supply-, environment- and demand-based policy tools, while policy ratings with strengths and weaknesseswere analyzed according to the Policy Modeling Consistency (PMC) index model.

**Results:**

LTCI policies of the 29 pilot cities all showed that the use of environment-based policy tools accounted for more than 70%, and supply- and demand-based policy tools ranged from 10 to 16%. The mean of the PMC index for the 79 policies was 7.701, with low scores across variables such as policy timeliness, policy level, and incentive and constraint, and an overall policy rating of good. Among these, the policies issued by the second batch of pilot cities had the highest PMC index value of 7.781.

**Conclusion:**

Pilot cities were over-utilizing environment-based policy tools and under-utilizing supply- and demand-based policy tools, which can be attributed to the reliance on environmental shaping during the policy pilot phase. LTCI policies of pilot cities can promote the development and improvement of the LTCI system, but there is still room for refinement in terms of policy authority, timeliness, coverage, and incentive measures. LTCI policies of the second batch of pilot cities were rated relatively higher, with higher-rated policies making more even use of various policy tools.

## 1 Introduction

The problem of population aging in China is becoming increasingly serious, with a growing number of the ﻿older adults and disabled ﻿older adults (1). According to data from the National Bureau of Statistics 2025, by the end of 2024, the population aged over 60 in China was 310 million, with those aged over 65 numbering 220 million, representing 22.0 and 15.6% of the total population, respectively (2). Meanwhile, the results of China’s fifth survey on the living conditions of the ﻿older adults showed that in 2021, among the population aged over 60, 42.7% rated their health as very good or relatively good, while 11.6% faced difficulties in self-care or were unable to care for themselves (3). The degree of disability and semi-disability rises rapidly with age, with the proportion of disability and semi-disability aged over 90 years being nine times higher than that aged 60–64 years (4). Although the disability rate of China’s ﻿older adults population has shown a downward trend in recent years with the improvement of education and accessibility to medical services, it will continue to rise rapidly soon with the increase in life expectancy. At the same time, the miniaturization of the family structure and the weakening of the family’s caregiving function for the ﻿older adults have made how to address “the huge demand for ﻿older adults care and security of care service supply” an issue of widespread concern to society (5).

Population aging, insufficient resources for home-based care and increasing costs of long-term care (LTC) are common issues faced by countries worldwide (6). World Social Report 2023 issued by the United Nations noted that changes in aging patterns have led to a continuous increase in demand for LTC, placing emphasis on how to integrate resources to provide safeguards for the disabled ﻿older adults and their families (7). It is difficult to solve the contradiction between the demand and supply of ﻿older adults care through market exchange mechanisms or social autonomy mechanisms, and the government should take the initiative to take part in solving the contradiction, with consideration of the overall interests of the society (8). The current degree of aging in China has exceeded the level of economic development, and it is difficult for the ﻿older adults to pay for LTC costs, threatening the survival rights of the ﻿older adults (9). This reinforces the need for the Chinese Government, as the main carrier of public power, to address the issue of LTC for the ﻿older adults. International practical experience has shown that the establishment and standardized implementation of the long-term care insurance (LTCI) system is an effective measure for the government to address the above issues, and to provide LTC services for the senior and disabled ﻿older adults through the systematic arrangement of risk diversification and transfer in advance, and timely compensation for the loss in the aftermath (10). Most developed countries and regions have actively established effective LTCI systems, which currently operate under three primary models.

Firstly, the social LTCI system is based on shared responsibilities among individuals, enterprises, and the government, with representation by Germany, Japan, and South Korea. Germany pioneered the establishment of such a system, implementing it in 1995. Since then, it has provided professional care services to the ﻿older adults population by integrating resources for retirement, medical care, and nursing, and paid for through insurance funds (11, 12). Japan stands as the nation with the most mature LTCI system among numerous countries sharing a similar cultural background and demographic structures with China. Established in 2000, Japan’s LTCI system has undergone six rounds of reform. Through macro-level institutional improvements, such as strengthening governmental oversight, prioritizing preventive care, and encouraging home-based ﻿older adults care, alongside micro-level refinements including premium increases, adjustments to personal co-payment rates, and improvements in care workers’ remuneration, Japan’s LTCI system has gained widespread public acceptance (13, 14). South Korea introduced its LTCI system in 2008, establishing a framework characterized by high universality and generosity, with a strong focus on supporting low-income groups and featuring multi-party cost-sharing arrangements (15, 16). Germany, Japan and South Korea have all established LTCI systems through a “legislation-first” approach, integrating LTCI into their social insurance frameworks, and stipulating the scope of coverage, funding mechanisms and benefit levels for such insurance.

Secondly, the market-led commercial LTCI system, represented by the United States. Originating in the 1970s, the commercial LTCI system in the United States has matured considerably. In particular, hybrid LTCI combining multiple insurance types now dominates the market, largely meeting the increasingly diversified and personalized LTC needs of the ﻿older adults (17, 18). Thirdly, welfare-based LTCI systems, representing the state, are found in numerous European nations. In countries such as the United Kingdom, Sweden, and Denmark, LTCI funding primarily derives from government taxation. Its purpose lies chiefly in safeguarding and meeting the care needs of the disabled ﻿older adults population, with benefit levels determined according to governmental policy objectives (19). The successful experiences of these developed nations, particularly the effective practices of their social LTCI systems, can offer valuable insights and inspiration for the Chinese policy framework.

The Chinese Government has gradually established and improved the LTCI system through policy formulation and implementation. *The Guidance Opinions on Launching the Pilot Long-Term Care Insurance System (“the Guidance”)* was issued in China in 2016, specifying 15 pilot cities for LTCI, which marked the official launch of the LTCI system at the national level (20). *The Guidance Opinions on Expanding the Pilot Long-Term Care Insurance System (“the Expanding-Guidance”)* was issued in China in 2020, clarifying the status of LTCI as independent insurance, while adding 14 new pilot cities for LTCI (21). Policies on LTC disability assessment standards and the management of designated care service organizations have since been issued, further standardizing and improving the LTCI system in China. The LTCI policy in China is issued for the ﻿older adults and the disabled, which involves LTC services and corresponding standardized management, fund-raising, and other policy knowledge and practical operation system, in order to cope with the reality of the transformation of ﻿older adults care from “family needs” to “socialized needs,” guarantee the survival rights of the ﻿older adults and their quality of life, and improve the system of the aging society (22, 23).

With the pilot implementation of LTCI in China, studies on LTCI have also been progressively conducted. Studies on the LTCI policy in China have focused on the following aspects: (a) Exploring the formulation and model selection of the LTCI policy in China based on foreign experience. For instance, Yin et al. employed the social welfare policy analysis framework to compile and compare the LTCI systems of Germany and Japan, suggesting that LTCI in China could leverage the basic medical security system, commencing with coverage for severely disabled individuals before gradually evolving into an independently operated insurance system (24). Xiao et al. examined the redistributive and social investment effects of LTCI systems in Germany, Japan and South Korea based on developmental welfare theory, and pointed out that China should establish the LTCI system without discrimination based on status, realize mutual assistance and solidarity among all groups, while ensuring that the co-payment ratio is not excessively high and setting cash benefit options with caution (25). (b) Evaluating the implementation effect of the LTCI policy in terms of the health effect and medical fee control effect. Relevant studies have found that LTCI can reduce wasteful healthcare expenditure, enhance the physical and mental wellbeing of the ﻿older adults, and alleviate the financial burden on families (26–29). (c) Proposing corresponding optimization recommendations based on the analysis of policy content and specific implementation in LTCI pilot areas. For instance, Dai et al. drawing from policy texts, and comparing differences among pilot areas in terms of beneficiaries, funding arrangements, services, and oversight, put forward suggestions for optimization in expanding coverage, broadening funding channels, enhancing benefit levels, and improving fund management (30). Xie et al. pointed out that during the pilot phase of the LTCI system in China, issues such as low coverage levels, the absence of an independent funding mechanism, and inconsistent assessment criteria have emerged, requiring improvements to the system’s operation in terms of the insured population, funding arrangements, and assessment standards (31).

Recently, quantitative analysis of policy texts using policy tools has been progressively applied to the examination of various policies. On the one hand, this approach helps mitigate misjudgments arising from value preferences inherent in qualitative analysis. On the other hand, the judicious selection and scientific design of policy tools can facilitate the successful attainment of policy objectives, particularly during the policy formulation stage (32). Therefore, based on an analysis of national-level policies in China (see Supplementary Table S1 in Additional File 1), this study starts from policy tools to establish a two-dimensional policy analytical framework comprising policy tools (X dimension) and policy ratings (Y dimension). This framework is used to conduct quantitative analysis on the LTCI policy texts of two batches of pilot cities. Taking the issuance time of *the Guidance* and *the Expanding-Guidance* as the node, the policies of the first and second batches of pilot cities in the first phase (after the issuance of *the Guidance* in 2016 - before the issuance of *the Expanding-Guidance* in 2020) and the second phase (after the issuance of *the Expanding-Guidance* in 2020–2024) are comparatively analyzed. This study aims to examine the tool combinations employed in LTCI policies and their strengths and weaknesses, explore existing instrumental issues and developmental shortcomings within current LTCI policies, and thereby provide reference points and insights for refining the Chinese LTCI policy framework. The first and second batches of pilot cities for LTCI are illustrated in Table 1.

**Table 1:** Distribution of the first and second batches of LTCI pilot cities in China.

Pilot batch	Pilot cities
The first batch	Chengde, Hebei; Changchun, Jilin; Qiqihar, Heilongjiang; Shanghai; Nantong, Jiangsu; Suzhou, Jiangsu; Ningbo, Zhejiang; Anqing, Anhui; Shangrao, Jiangxi; Qingdao, Shandong; Jingmen, Hubei; Guangzhou, Guangdong; Chongqing; Chengdu, Sichuan; Shihezi, Xinjiang
The second batch	Shijingshan, Beijing; Tianjin; Jincheng, Shanxi; Hohhot, Inner Mongolia; Panjin, Liaoning; Fuzhou, Fujian; Kaifeng, Henan; Xiangtan, Hunan; Nanning, Guangxi; Qianxinan Buyi and Miao Autonomous Prefecture, Guizhou; Kunming, Yunnan; Hanzhong, Shaanxi; Gannan Tibetan Autonomous Prefecture, Gansu; Urumqi, Xinjiang

## 2 Methods

### 2.1 Data sources and collection

This study aimed to evaluate and analyze LTCI policies of the first and second batches of pilot cities. Based on the principles of authoritativeness and comprehensiveness, relevant policies were searched and selected from the official government websites and other relevant websites, using the keywords “long-term care insurance” and “LTCI.” The selection criteria for the policies were: (a) issued by the relevant administrative departments of the first and second batches of pilot cities and based on national policies as a guide; (b) the main content around the establishment and implementation of the LTCI system and the expansion of its promotion; (c) issued from 2016 to 2024; (d) including departmental regulations, circulars, opinions, etc., excluding bulletins and approvals. A total of 79 policies were collected and screened for final inclusion in the analysis (see Supplementary Table S2 in Additional File 1), and the time of issuance of the two policies, *the Guidance and the Expanding-Guidance*, was used as the node, with 35 policies issued by the first batch of pilot cities in the first phase and 14 in the second phase, and 30 policies issued by the second batch of pilot cities all in the second phase.

### 2.2 Study design and analytical framework

Based on the specific content and characteristics of the LTCI policy, this study constructed the analytical framework of the LTCI policy from two dimensions: policy tools (X dimension) and policy ratings (Y dimension).

**Figure 1:**
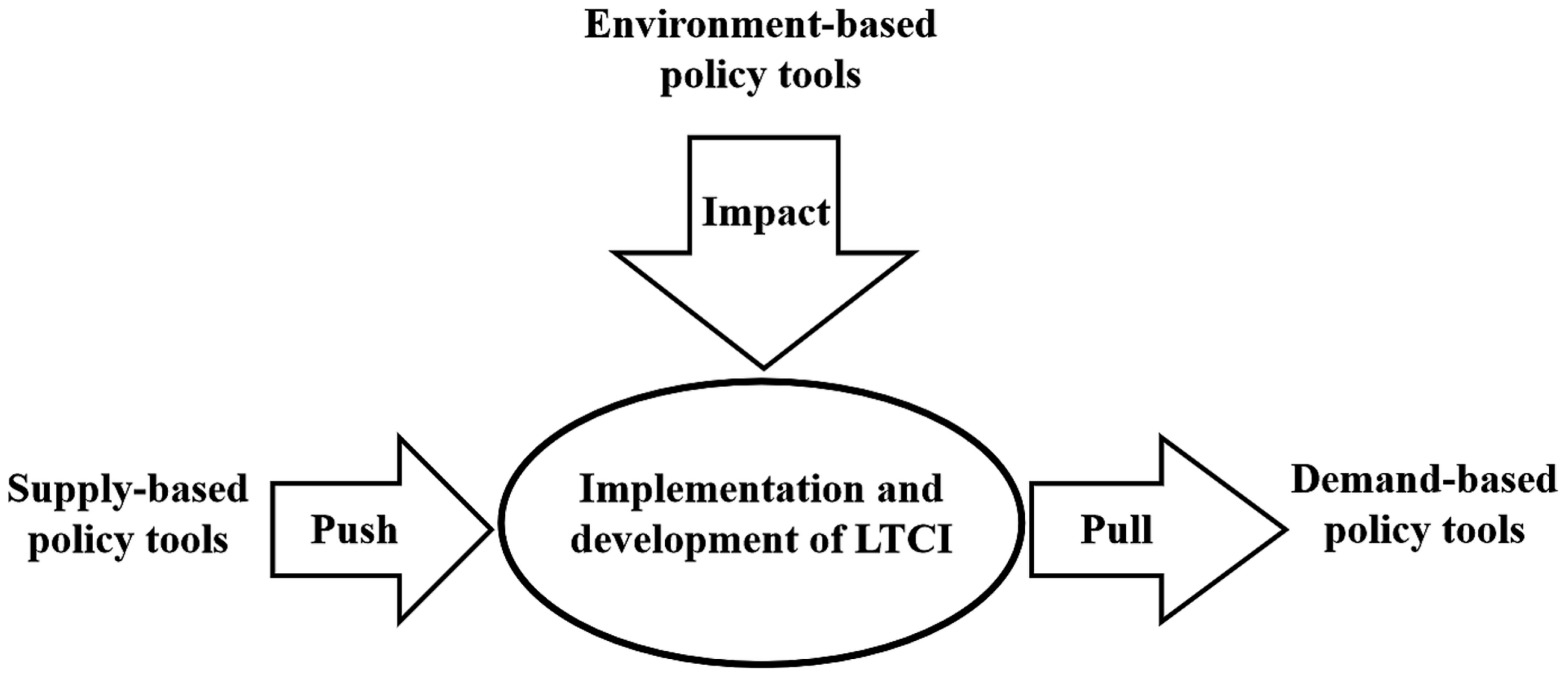
Policy tools for the implementation and development of LTCI.

Policy tools are important means for the government to achieve policy objectives, and are the paths and ways to translate substantive objectives into specific behaviors, with the choice and application of policy tools being an important bridge connecting policy objectives and policy outcomes (33). Policy tools play an important role in implementing and developing the LTCI policy, which in turn promotes the equitable and effective provision of LTC services. The policy tools were classified by Rothwell and Zegweld into three categories: supply-based, demand-based, and environment-based, depending on the subject of influence (34). This classification is multidimensional and comprehensive, attaches importance to the government’s guiding function, and weakens the mandatory characteristics of policy tools, which is widely used in policy analyses of ﻿older adults care services, healthcare, and social insurance (34–36). Given the applicability of this classification and the achievements of the studies in related fields, this study classified the policy tools into supply-based tools, environment-based tools, and demand-based tools. Supply-based policy tools are used by the government to promote the implementation of the LTCI policy in various aspects with the help of infrastructure construction, technology support, talent development, information support, and financial support. Environment-based policy tools indirectly and implicitly influence the implementation of the LTCI policy through the penetration of external factors, which provide a favorable development environment for the implementation of LTCI by the government through tax incentives, strategic measures, regulatory control, institution building, target planning, and policy publicity. Demand-based policy tools refer to the efforts made by the government to stimulate the demand for LTCI by taking measures such as government purchasing, policy subsidies, service outsourcing, market cultivation, and demonstration pilots, to pull forward the healthy development of LTCI. Figure 1 and Table 2 present the details.

**Table 2:** Classification and meaning of LTCI policy tools.

Type of policy tools	Name of policy tools	Meaning of policy tools
Supply-based	Infrastructure construction	Providing infrastructural safeguards for the implementation and development of LTCI through the construction of hardware facilities, such as professional ﻿older adults care organizations, health care organizations
	Technology support	Providing technology support to professional older adults care organizations, and health care organizations for the implementation of LTCI
	Talent development	Strengthening the nursing workforce and training nursing practitioners to provide professional labor support for the implementation of LTCI
	Information support	Providing the integrated and intelligent information platform for LTCI through the construction of the LTCI information database and information system
	Financial support	Providing financial support for the implementation and development of LTCI, such as financial investment, donation acceptance
Environment-based	Tax incentives	Creating a favorable tax environment for the implementation and development of LTCI through financial benefits, tax incentives, etc.
	Strategic measures	Adopting a series of specific measures, means and methods to promote the implementation and development of LTCI, such as improving the organization, division of labor and coordination, supervision and evaluation
	Regulatory control	Regulating various aspects of the implementation of LTCI through the formulation of regulations, systems and industry standards, to create a favorable environment under the rule of law for the development of LTCI
	Institution building	Improving the working methods of LTCI and standardizing the management criteria of the industry through the establishment of relevant institutions and mechanisms
	Target planning	Formulating overall targets, plans, programs, guidelines, and principles for the development of LTCI
	Policy publicity	Publicizing and promoting LTCI
Demand-based	Government purchasing	Using financial resources to purchase products and services related to LTCI
	Policy subsidies	Providing subsidies for the individual contributions of residents, preferential policies for the disadvantaged
	Service outsourcing	Commission third-party commercial insurance companies and other social forces to participate in LTCI administration services
	Market cultivation	Taking the lead in market cultivation efforts to promote LTCI development.
	Demonstration pilots	Promoting the effective implementation and stable development of LTCI through the construction of LTCI pilot projects and the establishment of typical and replicable pilot demonstration models

**Figure 2:**
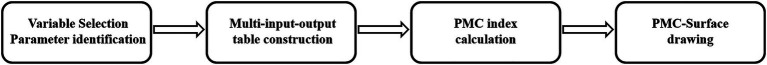
Analytical flowchart of the PMC index model.

The PMC index model was proposed by Ruiz-Estrada et al. as a policy evaluation tool based on the Omnia Mobilis hypothesis. It combines traditional text mining methods with advanced mathematical tools to construct a quantitative evaluation model, focusing on the raw data within policy texts, with the aim of incorporating relevant variables as comprehensively as possible (37, 38). The function of the PMC index model is, on the one hand, to evaluate the internal consistency of an individual policy, and on the other hand, to reflect the overall evaluation and specifics of an individual policy through the PMC index and PMC-surface (39). The lower the score on the PMC index, the more concave the PMC-surface becomes, and the lower the score on an individual indicator, the more pronounced the concavity of the PMC-surface appears within that indicator’s dimension, which reflects both the overall policy situation and variations across different aspects. The PMC index model is one of the more advanced policy evaluation models currently available. It enables a relatively objective analysis of the internal strengths and weaknesses of a policy across multiple dimensions, and overcomes the issues of subjectivity and ambiguity present in previous policy evaluations. It has been widely applied in policy evaluations within the fields of healthcare and ﻿older adults care (40, 41). The PMC index model can be used to quantify and objectively analyze policy texts by constructing a set of evaluation indicators and balancing each indicator using binary without limiting the number and weight of the indicators, and according to the results of the analyses, policies can be classified into four ratings of perfect, good, acceptable and poor (42, 43). The PMC index model provides a more operational, objective and applicable tool for evaluating LTCI policies. Based on the construction of evaluation indicators, this study calculated the PMC index to assign ratings to each policy. According to the mean of each indicator, PMC-surface chats were plotted to analyze the overall status of 79 LTCI policies, as well as the internal consistency, strengths and weaknesses of policies across different phases. Figure 2 illustrates the specific steps of the PMC index model, and this study was conducted in accordance with these steps.

Firstly, with reference to the classification of variables proposed by Ruiz Estrada and the results of existing studies (44–46), and taking into account the specific content characteristics of the policy, 10 first-level variables and 46 s-level variables for the evaluation of 79 LTCI policies were identified, as detailed in Table 3. Secondly, the multi-input–output table is a data analysis framework for quantitatively calculating individual variables through multiple dimensions. In this study, the first- and second-level variables mentioned above were put into the multi-input–output table, as detailed in Table 4, and each variable was given the same weight and assigned a value using binary (0, 1). Further, the PMC index was calculated for each LTCI policy, which included the following aspects (47): (a) the second-level variables were assigned values according to Equations 1, 2; (b) the value of each first-level variable was calculated according to Equation 3, which was between 0 and 1; (c) the PMC index of each LTCI policy was calculated according to Equation 4, and the criteria for classifying the policies according to the value of the PMC index were 9 to 10 (perfect), 7 to 8.999 (good), 5 to 6.999 (acceptable), 0 to 4.999 (poor) (38, 48). Finally, the PMC-surface for the LTCI policy was drawn by calculating the PMC matrix. The first-level variable X_10_ did not have any second-level variables, and each policy had a value of 1 in this variable. Considering the symmetry of the matrix and the balance of the surface, the first-level variable X_10_ was removed from the PMC-surface drawing, and a matrix of size 3*3 was formed by the other 9 first-level variables. The formula for the matrix is shown in Equation 5.


(1) X∼N[0,1]

(2) X={XR:[0∼1]}

(3) $$X_{t}(\sum_{j=1}^{n}\frac{X_{\mathrm{tj}}}{T(X_{\mathrm{tj}})}),t=1,2,3,4,5,6,7,8,9,10,\cdots ,\infty$$

**Table 3:** The evaluation variables and criteria for the LTCI policy.

First-level variable	Second-level variable	Evaluation criteria for second-level variable
Policy nature (X_1_)	Prediction (X_1-1_)	Whether it reflects predictions and forward-looking elements such as the gradual expansion of LTCI coverage, industrial development, and optimization of the talent structure, with yes 1, no 0.
	Recommendation (X_1-2_)	Whether it provides comments or recommendations on the development of LTCI, with yes 1, no 0.
	Feedback (X_1-3_)	Whether there is a corresponding feedback channel for problems, with yes 1, no 0.
	Supervision (X_1-4_)	Whether there is a corresponding supervision method, with yes 1, no 0.
	Description (X_1-5_)	Whether it involves details of the scope of participation, financing, disability assessment, treatment guarantee, fund management, service management, supervision and assessment, etc., with yes 1, no 0.
	Orientation (X_1-6_)	Whether it embodies the concept and value orientation of people-centered services, with yes 1, no 0.
Policy timeliness (X_2)_	Long-term (X_2-1_)	Whether the impact effectiveness is 5 years and above, with yes 1, no 0.
	Medium-term (X_2-2_)	Whether the impact effectiveness is 3–5 years, with yes 1, no 0.
	Short-term (X_2-3_)	Whether the impact effectiveness is 1–3 years, with yes 1, no 0.
	Within the year (X_2-4_)	Whether the impact effectiveness is less than 1 year, with yes 1, no 0.
Policy level (X_3_)	Administrative/local legislation (X_3-1_)	Whether it is administrative legislation or local legislation, with yes 1, no 0.
	Departmental/local regulation (X_3-2_)	Whether it is a departmental regulation or local government regulation, with yes 1, no 0.
	Normative document (X_3-3_)	Whether it is a normative document, with yes 1, no 0.
	Industry regulation (X_3-4_)	Whether it is an industry regulation, with yes 1, no 0.
Policy subject (X_4_)	The government (X_4-1_)	Whether it involves the government, with yes 1, no 0.
	Insurance companies (X_4-2_)	Whether it involves insurance companies, with yes 1, no 0.
	Professional care organizations (X_4-3_)	Whether it involves professional care organizations, with yes 1, no 0.
	Social organizations (X_4-4_)	Whether it involves social organizations, with yes 1, no 0.
	Third-party assessment organizations (X_4-5_)	Whether it involves third-party assessment organizations, with yes 1, no 0.
	The public (X_4-6_)	Whether it involves the public such as urban employees and urban and rural residents, with yes 1, no 0.
	Beneficiaries (X_4-7_)	Whether it involves beneficiaries such as the disabled, with yes 1, no 0.
Policy objective (X_5_)	Care guarantee (X_5-1_)	Whether it involves the provision of care guarantee for the disabled, with yes 1, no 0.
	Financial compensation (X_5-2_)	Whether it involves the provision of financial protection or compensation for the disabled, with yes 1, no 0.
	Pilot extension (X_5-3_)	Whether it involves the pilot extension of LTCI for the ﻿older adults with yes 1, no 0.
Policy content (X_6_)	Disability assessment (X_6-1_)	Whether it involves disability assessment, with yes 1, no 0.
	Funding (X_6-2_)	Whether it involves funding, with yes 1, no 0.
	Service provision (X_6-3_)	Whether it involves service provision, with yes 1, no 0.
	Treatment payment (X_6-4_)	Whether it involves treatment payment, with yes 1, no 0.
	Standardized management (X_6-5_)	Whether it involves the standardized management of funds, services, administration, etc., with yes 1, no 0.
	Organization and implementation (X_6-6_)	Whether it involves supporting measures and operational mechanisms for LTCI, etc., with yes 1, no 0.
Incentive and constraint (X_7_)	Financial inputs (X_7-1_)	Whether it includes financial inputs, with yes 1, no 0.
	Tax incentives (X_7-2_)	Whether it includes tax incentives, with yes 1, no 0.
	Financial support (X_7-3_)	Whether it includes financial support, with yes 1, no 0.
	Talent training (X_7-4_)	Whether it includes talent training, with yes 1, no 0.
	Government purchasing (X_7-5_)	Whether it includes government purchasing, with yes 1, no 0.
	Information support (X_7-6_)	Whether it includes information support, with yes 1, no 0.
	Institutional guarantee (X_7-7_)	Whether it includes institutional guarantees such as institutional mechanism, with yes 1, no 0.
Policy function (X_8_)	Macro design (X_8-1_)	Whether the institutional design or overall planning of LTCI is carried out from a macro or holistic perspective, with yes 1, no 0.
	Supervisory constraints (X_8-2_)	Whether it involves the supervision and constraints of the implementation of LTCI, with yes 1, no 0.
	Normative guidance (X_8-3_)	Whether it involves the normative guidance of institutional details such as disability assessment, treatment payment, etc., with yes 1, no 0.
	Encouragement and motivation (X_8-4_)	Whether it contains corresponding measures to promote the development of LTCI such as encouragement and motivation, with yes 1, no 0.
	Service optimization (X_8-5_)	Whether it involves the improvement of the LTCI system and service optimization, etc., with yes 1, no 0.
Policy evaluation (X_9_)	Well founded (X_9-1_)	Whether there is sufficient foundation, such as national laws and legislations, central government documents, etc., with yes 1, no 0.
	Clear objectives (X_9-2_)	Whether it has clear objectives, such as implementing the people-centered development idea, institutional improvement, and disability care protection, etc., with yes 1, no 0.
	Detailed planning (X_9-3_)	Whether the policy plan is detailed, such as involving the management of disability level assessment, management of commissioned agencies, management of designated and agreed care service organizations, the scope of participation, financing, disability assessment, treatment guarantee, fund management, service management, supervision and assessment, etc., with yes 1, no 0.
	Scientific program (X_9-4_)	Whether the policy program is scientific and feasible, with yes 1, no 0.
Policy information disclosure (X_10_)		Whether the policy is public and transparent, with yes 1, no 0.

**Table 4:** Input–output table for the LTCI policy.

First-level variable	Second-level variable
X_1_	X_1-1_﻿, X_1-2_, X_1-3_, X_1-4_, X_1-5_, X_1-6_
X_2_	X_2-1_, X_2-2_, X_2-3_, X_2-4_
X_3_	X_3-1_, X_3-2_, X_3-3_, X_3-4_
X_4_	X_4-1_, X_4-2_, X_4-3_, X_4-4_, X_4-5_, X_4-6_, X_4-7_
X_5_	X_5-1_, X_5-2_, X_5-3_
X_6_	X_6-1_, X_6-2_, X_6-3_, X_6-4_, X_6-5_, X_6-6_
X_7_	X_7-1_, X_7-2_, X_7-3_, X_7-4_, X_7-5_, X_7-6_, X_7-7_
X_8_	X_8-1_, X_8-2_, X_8-3_, X_8-4_, X_8-5_
X_9_	X_9-1_, X_9-2_, X_9-3_, X_9-4_
X_10_	

*t* is the first-level variable, *j* is the second-level variable, and *T (X_tj_)* is the number of second-level variables under the first-level variable.


(4) $$\mathrm{PMC}=\{\begin{array}{c} X_{1}(\sum_{i=1}^{6}\frac{X_{1i}}{6})+X_{2}(\sum_{j=1}^{4}\frac{X_{2j}}{4})+X_{3}(\sum_{k=1}^{4}\frac{X_{3k}}{4})+\\ X_{4}(\sum_{l=1}^{7}\frac{X_{4l}}{7})+X_{5}(\sum_{m=1}^{3}\frac{X_{5m}}{3})+X_{6}(\sum_{n=1}^{6}\frac{X_{6n}}{6})+\\ X_{7}(\sum_{o=1}^{7}\frac{X_{7o}}{7})+X_{8}(\sum_{p=1}^{5}\frac{X_{8p}}{5})+X_{9}(\sum_{r=1}^{4}\frac{X_{9r}}{4})+X_{10} \end{array}\}\;$$

(5) $$\mathrm{PMC}-\text{Surface}=(\begin{array}{c} X_{1}\;\;X_{2}\;\;X_{3}\\ X_{4}\;\;X_{5}\;\;X_{6}\\ X_{7}\;\;X_{8}\;\;X_{9} \end{array})$$

**Figure 3:**
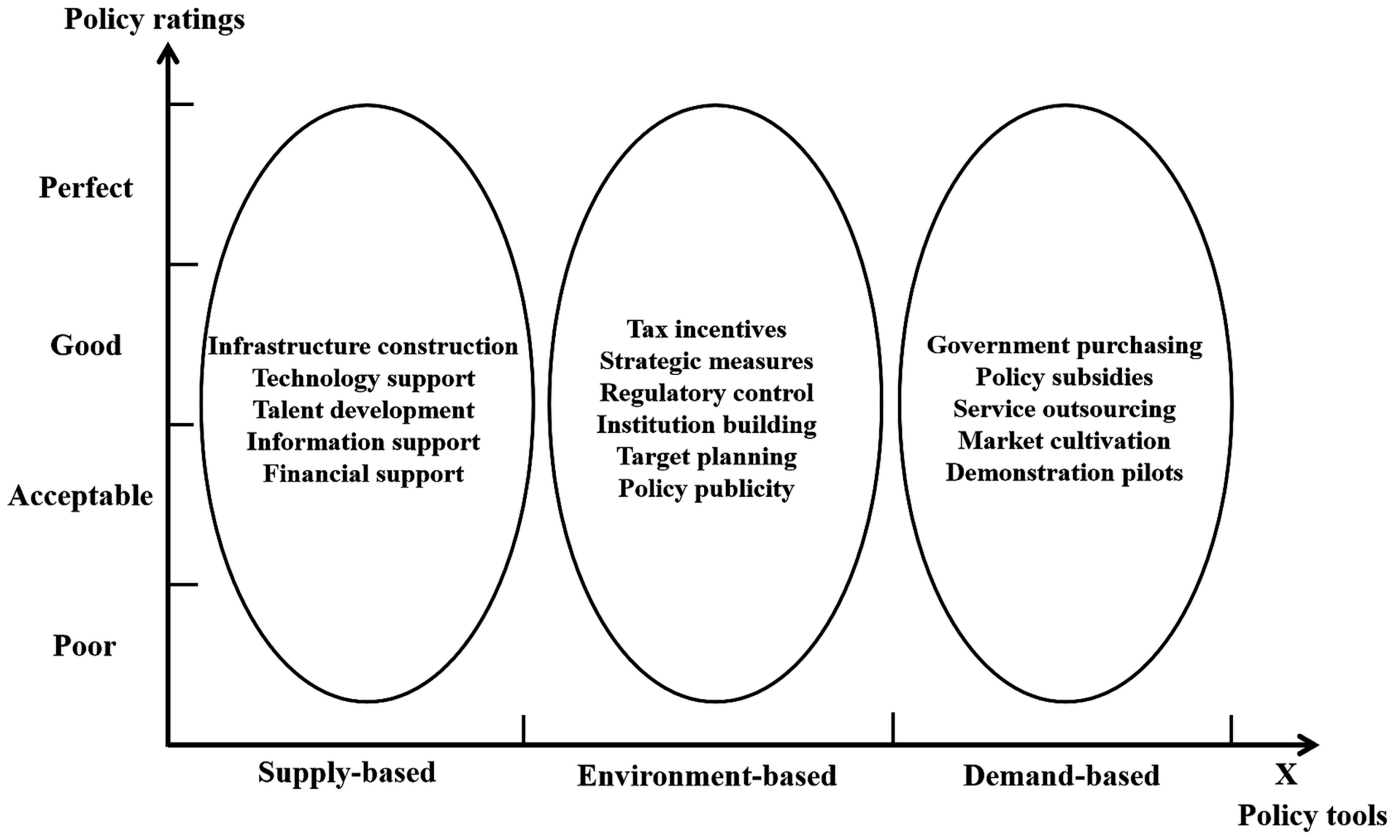
Analytical framework for the LTCI policy.

The two-dimensional analytical framework constructed by combining policy tools (X dimension) and policy ratings (Y dimension) is shown in Figure 3.

### 2.3 Analytical methods

Taking the 79 LTCI policies of the first and second batches of pilot cities as the sample for analysis, the specific contents of the policy texts were coded, categorized, counted for their frequency of use, and sorted and numbered according to region and chronological order. First, the policies were coded according to “policy number-policy chapter-specific unit of analysis,” for example, the code “1–1-1” indicated the first analytical unit in the first chapter of the first policy. Second, the policy codes were categorized according to the analytical framework of the policy tools, and the frequency of each categorized unit was counted﻿.

## 3 Results

### 3.1 Results of the policy tools analysis

A total of 2,914 coding units were obtained from the text coding of 79 LTCI policies in this study. The results of the analysis showed that the pilot cities in China preferred to combine three types of policy tools: supply-based, environment-based, and demand-based in their policy formulation. However, the degree to which these three types of policy tools were used varied significantly, revealing a pronounced structural unevenness. At all phases, the frequency of using the environment-based policy tools was the highest, accounting for more than 70% of the total, and under this policy tool, strategic measures were utilized the most, accounting for more than 50% of the total. Subsequently, the use of institution building and target planning was relatively common, each accounting for approximately 7%, while tax incentives and policy publicity were employed less frequently, each representing around 1%. This indicated variations in the use of the sub-policy tools within the environment-based policy tools, with the government showing a clear preference for implementing detailed arrangements for LTCI operations and placing greater emphasis on its development targets and principles. Conversely, there was a marked deficiency in investment financing and publicity guidance for LTCI.

Across all phases, the frequency of using supply-based policy tools ranged between 10 and 16%. The frequency of employing sub-policy tools, including infrastructure construction, talent development, information support, and financial support, all remained below 5%. Technology support was the least used, with a frequency of less than 1%. This indicated that policy support for the hardware and software facilities of ﻿older adults care institutions, as well as for professional nursing teams, was relatively limited. This situation was detrimental to the optimal allocation of care resources.

Demand-based policy tools maintained a frequency of use at around 10% across all phases. Among these, sub-tools such as policy subsidies and market cultivation were only used around 1%, while government purchasing was an even lower, at less than 1%. This was incompatible with the need for effective policy traction to stimulate demand during the initial phase of the LTCI system.

**Figure 4:**
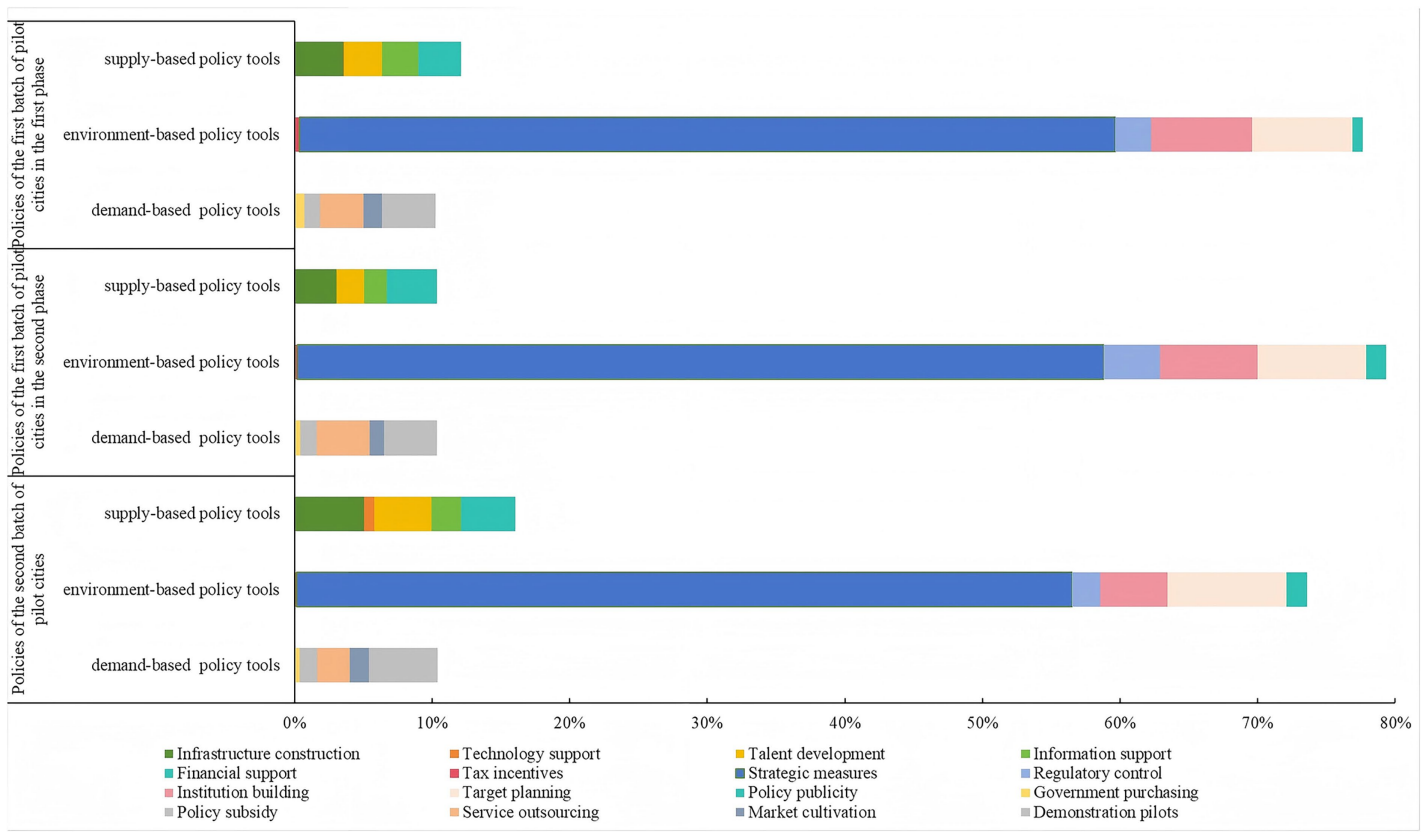
Comparison of the use of policy tools in different phases.

In terms of the different phases, the policies of the first batch of pilot cities in the second phase made the most use of environment-based policy tools, with a proportion of 79.310%, while the policies of the second batch of pilot cities made relatively less use of environment-based policy tools, with a proportion of 73.571%. Policies of the first batch of pilot cities in the second phase made the least use of supply-based policy tools, with a proportion of 10.345%, while policies of the second batch of pilot cities made relatively more use of supply-based policy tools, with a proportion of 16.026%. The proportion of demand-based policy tools used remained around 10% in all phases. Regarding sub-policy tools, within the environment-based policy tools, the percentage of strategic measures utilized decreased from 59.306 to 56.326%, the percentage of institutional building utilized decreased from 7.312 to 4.873%, while the percentage of policy publicity utilized increased from 0.739 to 1.500%. Within the supply-based policy tools, the use of infrastructure construction increased from 3.545 to 5.061%, talent development rose from 2.733 to 4.124%, and technology support was used significantly more times in the policies of the second batch of pilot cities, reaching eight times. This indicates that the second batch of pilot cities for LTCI have employed policy tools more rationally. The specific situation of various policy tools at different phases is shown in Table 5﻿ and Figure 4.

**Table 5:** Distribution of LTCI policy tools.

Type of policy tools	Name of policy tools	Policies of the first batch of pilot cities in the first phase		Policies of the first batch of pilot cities in the second phase		Policies of the second batch of pilot cities	
		Frequency (times)	Percentage (%)	Frequency (times)	Percentage (%)	Frequency (times)	Percentage (%)
Supply-based	Infrastructure construction	48	3.545	15	3.043	54	5.061
	Technology support	1	0.074	0	0.000	8	0.750
	Talent development	37	2.733	10	2.028	44	4.124
	Information support	36	2.659	8	1.623	23	2.156
	Financial support	42	3.102	18	3.651	42	3.936
	Total	164	12.112	51	10.345	171	16.026
Environment-based	Tax incentives	5	0.369	1	0.203	2	0.187
	Strategic measures	803	59.306	289	58.621	601	56.326
	Regulatory control	35	2.585	20	4.057	22	2.062
	Institution building	99	7.312	35	7.099	52	4.873
	Target planning	99	7.312	39	7.911	92	8.622
	Policy publicity	10	0.739	7	1.420	16	1.500
	Total	1,051	77.622	391	79.310	785	73.571
Demand-based	Government purchasing	10	0.739	2	0.406	4	0.375
	Policy subsidies	15	1.108	6	1.217	14	1.312
	Service outsourcing	43	3.176	19	3.854	25	2.343
	Market cultivation	18	1.329	5	1.014	15	1.406
	Demonstration pilots	53	3.914	19	3.854	53	4.967
	Total	139	10.266	51	10.345	111	10.403
Total		1,354	100	493	100	1,067	100

### 3.2 Results of PMC index calculations

The PMC index of each LTCI policy was calculated based on the PMC index model, and each policy was ranked and rated accordingly. The results of the PMC index for selected LTCI policies are demonstrated in Table 6, and the complete PMC index results for 79 LTCI policies can be found in Supplementary Table S3 in Additional File 1. The mean of the PMC index for the 79 policies was 7.701, the policy rating was good, and the mean of the variables X_1_ ~ X_10_ were 0.840, 0.307, 0.563, 0.808, 0.937, 0.924, 0.539, 0.873, 0.910, and 1. The mean of the seven variables, including policy nature, policy subject, policy objective, policy content, policy function, policy evaluation, and policy information disclosure, all were above 0.8. Compared with these, the mean of policy timeliness, policy level, and incentive and constraint were relatively low. Considering the specific variables, this showed that policy timeliness was relatively short, policy levels were predominantly normative documents and industry regulations, and the incentive and constraint effects of policies were not strong.

**Table 6:** Results of the PMC index for selected LTCI policies in China.

Policy number	X_1_	X_2_	X_3_	X_4_	X_5_	X_6_	X_7_	X_8_	X_9_	X_10_	PMC index	Ranking	Rating
1	0.833	0.250	0.750	0.857	1	1	0.857	1	1	1	8.547	12	good
2	0.833	0.250	0.500	0.857	1	0.833	0.714	1	1	1	7.987	40	good
3	0.833	0.250	0.500	0.857	1	1	0.286	1	1	1	7.726	51	good
4	1	0.250	0.750	1	1	1	0.714	1	1	1	8.714	4	good
5	0.500	0.250	0.500	0.571	1	1	0.143	0.400	1	1	6.364	72	acceptable
…	…	…	…	…	…	…	…	…	…	…	…	…	…
16	1	0.500	0.500	1	1	1	1	1	1	1	9	2	perfect
17	0.500	0.250	0.500	0.857	1	0.667	0.286	0.400	0.500	1	5.960	76	acceptable
18	0.500	0.250	0.500	0.857	1	1	0.429	0.400	0.500	1	6.436	71	acceptable
19	0.833	0.500	0.500	0.857	1	0.833	1	1	1	1	8.523	15	good
20	1	0.500	0.750	1	1	1	0.857	1	1	1	9.107	1	perfect
…	…	…	…	…	…	…	…	…	…	…	…	…	…
75	1	0.250	0.500	0.857	1	1	0.571	1	1	1	8.178	29	good
76	1	0.500	0.500	0.857	1	1	0.429	1	1	1	8.286	23	good
77	0.333	0.250	0.500	0.429	0.333	0.333	0.286	0.600	0.500	1	4.564	78	poor
78	1	0.250	0.500	1	1	1	0.571	1	1	1	8.321	22	good
79	1	0.500	0.500	1	1	1	0.571	1	1	1	8.571	8	good

**Figure 5:**
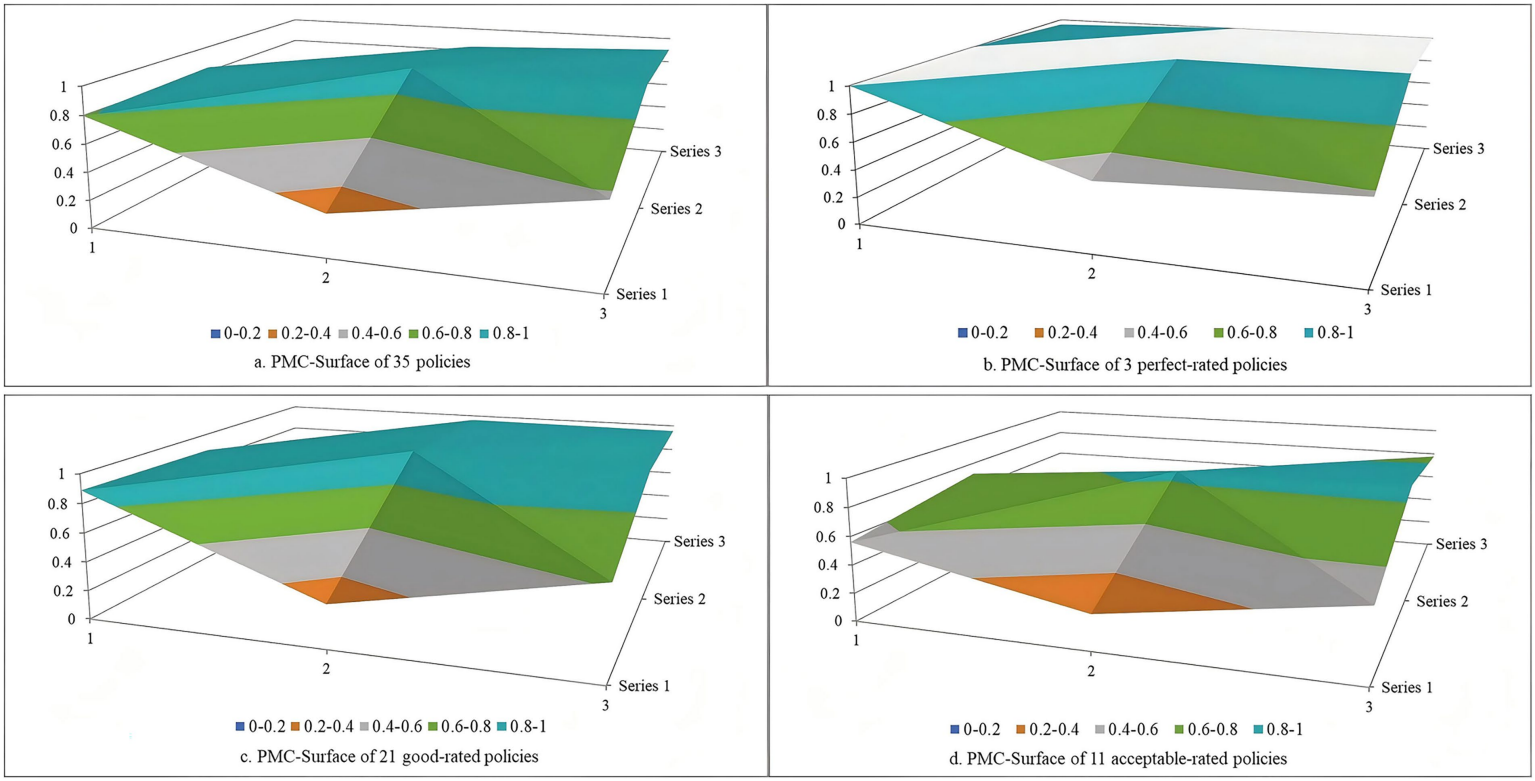
PMC-surface chats of policies of the first batch of pilot cities in the first phase.

For the 35 policies of the first batch of pilot cities in the first phase, the mean of the PMC index was 7.683, with the policy rating of good, and the mean of the variables X_1_ ~ X_10_ were 0.795, 0.300, 0.579, 0.832, 0.933, 0.919, 0.588, 0.834, 0.903, and 1. The mean of the PMC index for this phase was lower than that of the overall 79 policies with a mean of 7.701, primarily due to lower scores in the variables of policy nature and policy function. Among these 35 policies, policies 14, 16, and 20 were rated as perfect with values of the PMC index of 9, 9, and 9.107, respectively. 21 policies were rated as good, and 11 policies were rated as acceptable. The PMC-surface charts of LTCI policies with different ratings were constructed based on the values of the PMC index (Figure 5). The vertical coordinate indicated the values of the PMC index, and the horizontal coordinate corresponded to the different dimensions of X_1_ ~ X_9_. These 35 policies overall exhibited moderate concavity. While the concavity of three variables, including policy timeliness, policy level, and incentive and constraint, was large, the concavity of other variables was relatively small. This suggested that these three variables represent key areas for future policy optimization. Furthermore, the three policies rated as perfect demonstrated the least concave, while the 11 policies rated as acceptable exhibited the most concave.

**Figure 6:**
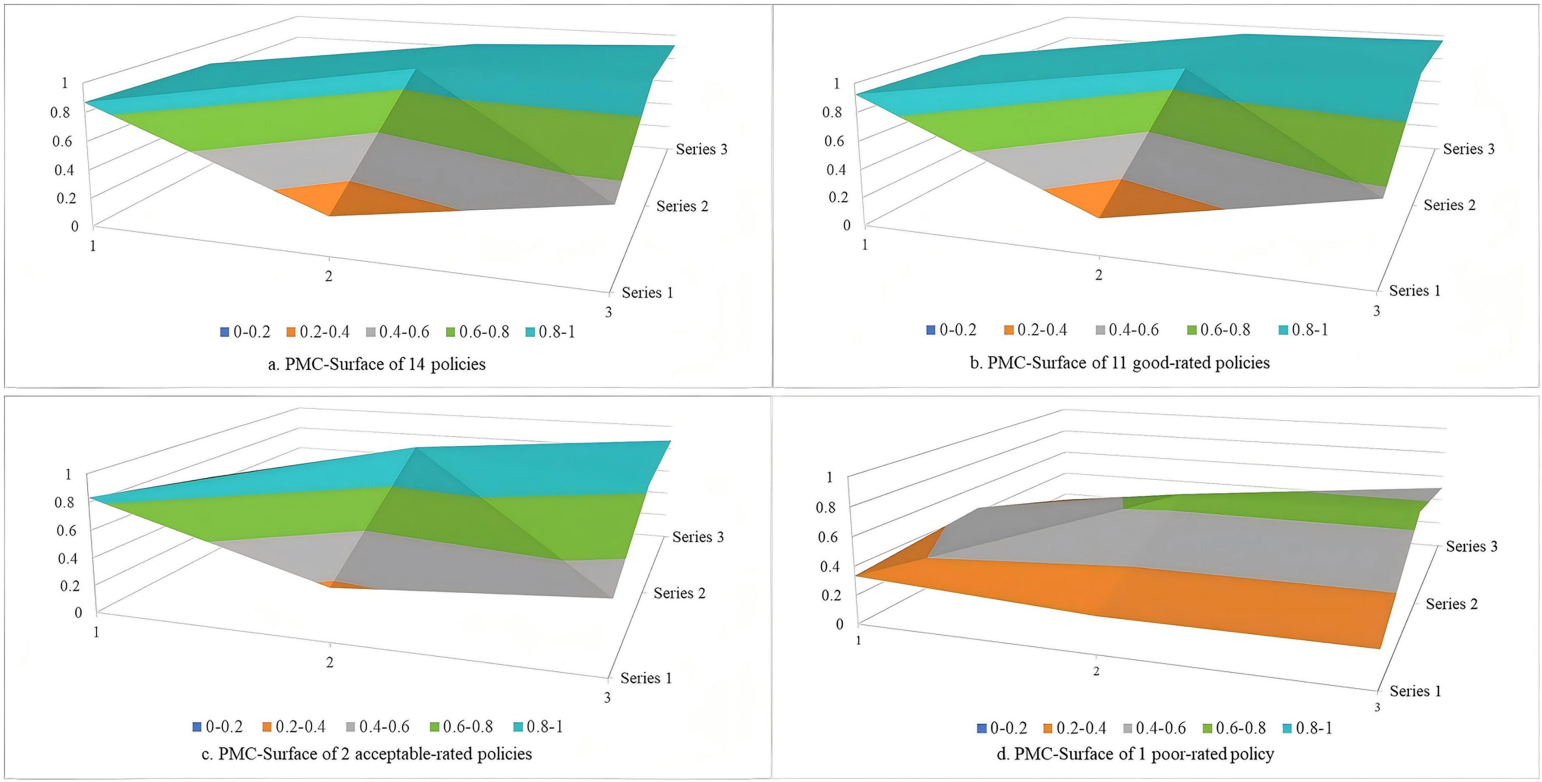
PMC-surface chats of policies of the first batch of pilot cities in the second phase.

For the 14 policies of the first batch of pilot cities in the second phase, the mean of the PMC index was 7.577, with the policy rating of good, and the mean of variables X_1_ ~ X_10_ were 0.869, 0.268, 0.536, 0.837, 0.905, 0.940, 0.480, 0.829, 0.914, and 1. At all stages, the 14 policies in this phase exhibited the lowest mean of PMC index, primarily manifested in the shortest policy timeliness, the lowest policy level, unclear policy objective and function, and limited effectiveness in incentive and constraint. Of these 14 policies, 11 were rated as good, and two policies, 36 and 38, were rated as acceptable, with PMC index values of 6.730 and 6.873, respectively. Policy 43 had a PMC index value of 4.439, with a rating of poor. The PMC-surface charts of LTCI policies with different ratings were constructed based on the values of the PMC index (Figure 6). These 14 policies overall were moderate concavity, with one poor-rated policy being unacceptable concavity.

**Figure 7:**
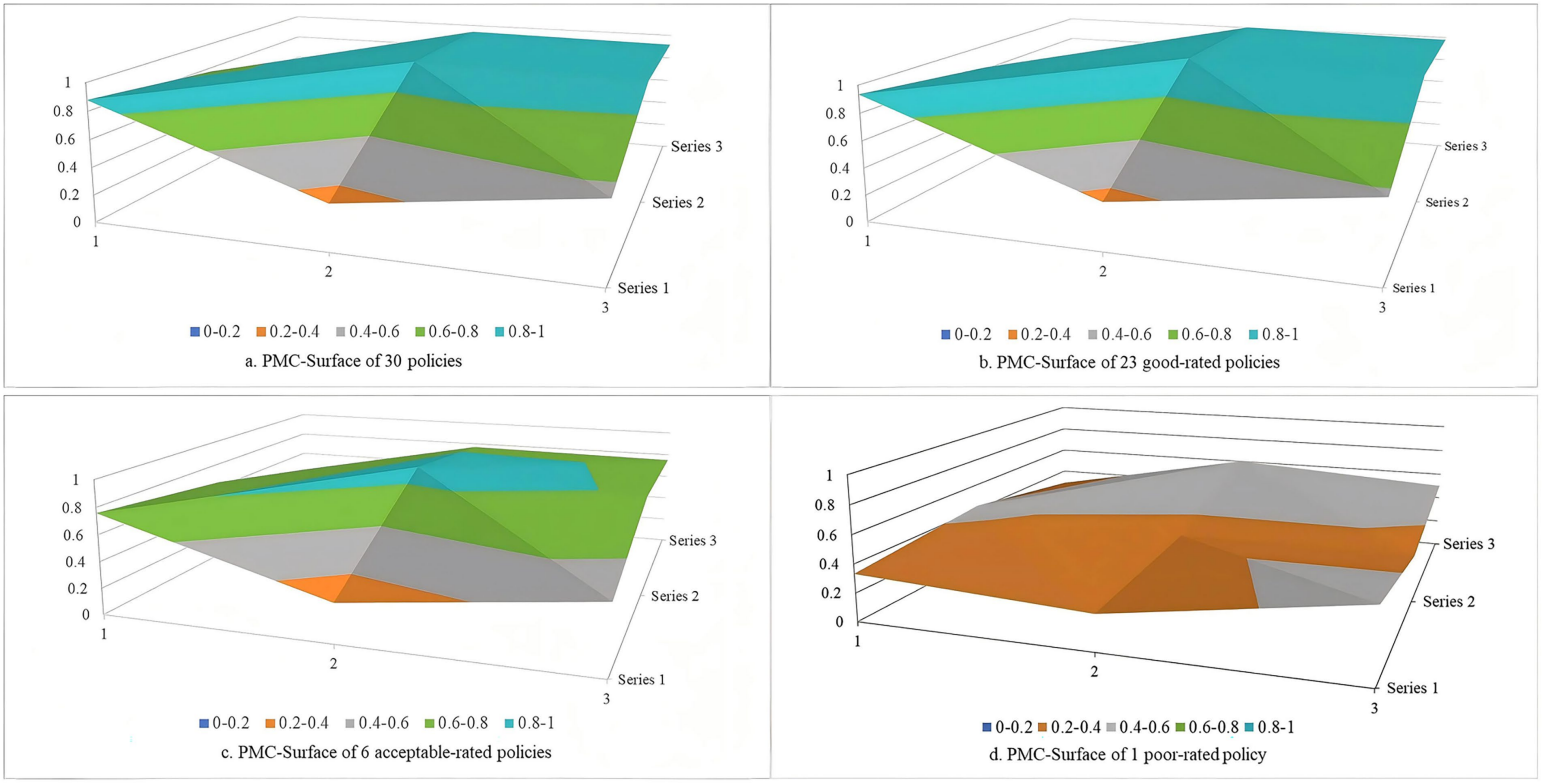
PMC-surface chats of policies of the second batch of pilot cities.

For the 30 policies of the second batch of pilot cities, the mean of the PMC index was 7.781, and the policies were rated as good. The means of variables X_1_ ~ X_10_ were 0.879, 0.333, 0.558, 0.766, 0.956, 0.922, 0.509, 0.940, 0.917, and 1. Across all phases, the 30 policies in this phase had the highest mean of PMC index, primarily due to high scores in the variables of policy nature, policy objective, and policy function, though the variable of policy subject scored relatively low. Among these 30 policies, 23 were rated as good and 6 were rated as acceptable. The PMC index value of policy 77 was 4.564, and the policy was rated as poor. The PMC-surface charts of LTCI policies with different ratings were constructed based on the values of the PMC index (Figure 7). These 30 policies were overall moderately concave, with one poor-rated policy exhibiting unacceptable concavity. Furthermore, significant concavity was observed across four variables, including policy timeliness, policy level, policy subject, and incentive and constraint, indicating that these four variables represent key areas for future policy optimization.

### 3.3 Results of the two-dimensional cross-analysis

Overall, policies with ratings of perfect, good, and acceptable had the most use of environment-based policy tools, relatively less use of supply-based policy tools, and the least use of demand-based policy tools. Policies with a rating of poor were used 10 times for environment-based policy tools, 3 times for demand-based policy tools, and no supply-based policy tools were used.

Specifically, only three policies from the first batch of pilot cities in the first phase were rated as perfect, with the most frequent use of environment-based policy tools, at 75 times, and supply- and demand-based policy tools, at 12 and 17 times, respectively. Among the policies with a rating of perfect, the frequency of using the environment-based policy tools was 6.25 and 4.41 times higher than that of the supply- and demand-based policy tools, respectively.

Among the policies rated as good, policies of the first batch of pilot cities in the first phase used supply-based policy tools the least (9.601%), while policies of the second batch of pilot cities used supply-based policy tools relatively more (13.215%). Policies of the first batch of pilot cities in the second phase used the environment-based policy tools the most (74.848%), and policies of the first batch of pilot cities in the first phase used environment-based policy tools relatively less (52.511%). Demand-based policy tools were used most (9.736%) in the policies of the first batch of pilot cities in the second phase and relatively less (6.869%) in the policies of the first batch of pilot cities in the first phase. The frequency of using environment-based policy tools for good-rated policies was about 5 to 7 times as frequent as supply- and demand-based policy tools, and only policies rated as good in the second batch of pilot cities used environment-based policy tools 4.52 times more frequently than supply-based policy tools.

Of the policies with a rating of acceptable, supply-based policy tools were not used in the policies of the first batch of pilot cities in the second phase, and were used relatively more in the policies of the second batch of pilot cities (2.812%). Environment-based policy tools were most used in the policies of the first batch of pilot cities in the first phase (19.572%) and relatively less used in the policies of the first batch of pilot cities in the second phase (3.857%). Demand-based policy tools were least used in the policies of the first batch of pilot cities in the second phase (0.609%) and relatively more used in the policies of the first batch of pilot cities in the first phase (2.142%). In this rating of policies, the frequency of using environment-based policy tools in the first batch of pilot cities in the first phase was 12 and 9.14 times more frequent than supply-based and demand-based policy tools, respectively, while in the second batch of pilot cities, the frequency of using environment-based policy tools was 4.67 and 7.78 times more frequent than supply-based and demand-based policy tools, respectively.

Among the policies with a rating of poor, both the policies of the first batch of pilot cities in the second phase and the policies of the second batch of pilot cities did not use supply-based policy tools, and the use of environment-based policy tools was similar, accounting for 0.609 and 0.656%, respectively. Only the policies of the second batch of pilot cities used demand-based policy tools, accounting for 0.281%.

Taking the above findings together, although policies across all ratings tended toward the use of environment-based policy tools, policies with higher ratings exhibited a relatively lower propensity for environment-based policy tools, with greater balance in the use of various policy tools. Policies of the second batch of pilot cities, being of a higher rating than those of the first batch, also demonstrated relatively balanced utilization of policy tools. Further details are presented in Supplementary Table S4 in Additional File 1.

## 4 Discussion

### 4.1 Uneven distribution within policy tools

Analysis of policy texts from pilot cities for LTCI revealed that three types of policy tools were employed at all stages. However, environment-based policy tools were utilized most frequently, particularly in relation to sub-policy tools such as strategic measures, institutional building, and target planning. Supply-based and demand-based policy tools saw a lower frequency of use, indicating insufficient leverage of policy push and pull effects. Overall, though the policy tool usage patterns remained similar across stages, the proportions applied to each policy tool exhibited certain variations. The specific discussion and analysis of policy tools is as follows.

The frequency of using environment-based policy tools was the highest at all phases, reflecting the reliance of the government on environment-based policy tools. Zhang, Wu et al. analyzed LTCI policies in pilot regions of western and central China, similarly finding that governments preferred to employ environment-based policy tools (49, 50). The LTCI policy has been in the pilot stage, and the early policy needs to provide a clear institutional arrangement and development environment with the help of environment-based policy tools (51). However, excessive reliance on such policy tools is likely to result in LTCI policies becoming hollow in substance and failing to be effectively implemented in practice. Additionally, among the sub-policy tools, the use of strategic measures has been particularly high, followed by the relatively high use of institution building and target planning. This reflects the need for a series of detailed working arrangements, strengthened institutional norms and guidance, and clear development objectives for the pilot phases of LTCI, to provide a good environment for the orderly development of LTCI work (52). Instead, it has been used less for policy publicity and tax incentives. On the one hand, this is not conducive to the public fully understanding LTCI, affecting the rate of popularization and participation in LTCI. On the other hand, this is not conducive to the access of social capital to the LTCI market, and unfavorable to the broadening of investment and financing channels of LTCI, which makes it difficult to unleash the vitality of the market for LTCI (53).

Supply-based policy tools have given insufficient push to LTCI, with relatively little investment in infrastructure construction, talent development, information support, financial support, and even less investment in technology support. Zhou et al. also observed similar findings in their analysis of the policy text for LTCI in Nantong, Jiangsu Province, where supply-based policy tools were employed less frequently, and the technology support sub-policy tool was not utilized at all (22). This can lead to a series of problems, such as the insufficient supply of LTC services, low service levels, and a lack of professional caregivers, which are more prominent in rural areas (54, 55). Li et al. similarly noted in their research that rural areas faced more limited resources, with shortages of care practitioners and substandard LTC services, resulting in severe challenges to meeting the LTC needs of the disabled ﻿older adults in rural communities (56). Furthermore, China depends excessively on public health insurance funds and financial subsidies to finance LTCI, and lacks a multi-channel financing mechanism, which affects the stable and sustainable supply of funds (57). Li et al. also noted in their research that limited financing options and overreliance on public health insurance funds posed challenges threatening the sustained and effective development of LTCI (56).

On the one hand, an insufficient supply of funds reduces the salary level of LTC practitioners and limits training opportunities, thus aggravating the wastage of LTC practitioners and making it difficult to ensure the quality of care services. On the other hand, insufficient supply of funds limits investment in technology, information, infrastructure and other resources. Studies have shown that the ﻿older adults have poor technological literacy and low acceptance of intelligent services, and the application of intelligent information technology plays an important role in improving the efficiency and quality of LTC services, so there is a need to increase investment in information, technology and other resources to promote the safe and convenient use of intelligent services (58).

The pull effect of demand-based policy tools in enhancing the accessibility of LTCI and activating the demand for service targets has not been effectively brought into play, with insufficient efforts to cultivate the market (59), which was similar to the findings of Zhou, Zhang, Wu, et al. (22, 49, 50). Pilot cities have rarely used policy subsidies and government purchasing of sub-policy tools, which is related to factors such as the expansion of the scale of the ﻿older adults with disability in China, the increase in the demand for care, the insufficient inflow of social capital, and the increase in financial pressure on the government (60). The insufficient use of demand-based policy tools has also led to the failure to effectively meet the current demand for LTC. The current LTCI coverage in China is limited, primarily targeting severely physically disabled individuals who have been disabled for more than 6 months, while moderately, mildly physically disabled and intellectually disabled individuals are largely excluded from coverage (27). Among the multiple pilot cities surveyed by Wang et al., only Qingdao included those with intellectual disability within the coverage scope of its LTCI system (61). Additionally, the level of LTC service quality is relatively low, making it difficult to meet the demand for personalized care services, and the satisfaction with daily living care demands remains inadequate. There is a need to enhance the levels of treatment for disabled individuals. Wu et al. similarly recommend expanding the coverage of LTCI services to foster a people-centered care environment and meet the needs of the demand-side market (62).

Among the policies in the first batch of pilot cities in the first phase, the use of environment-based policy tools accounted for 77.622%, and in the policies of the second phase, the use of environment-based policy tools increased slightly to 79.310%, which reflected the increase in the use of regulatory control and policy publicity. Based on the policy experience in the first phase, the implementation of LTCI should emphasize the standardized management of stakeholders and the enhancement of policy authority, and the acceptance of and participation in LTCI by the demand side should also be enhanced through strengthened policy publicity (63). Compared with the first batch of pilot cities, the second batch of pilot cities have slightly decreased the use of environment-based policy tools, which can be seen in the decrease in the proportion of strategic measures and institution building. The main reason is that the policy experience of the first batch of pilot cities can provide a reference for the policy formulation of the second batch of pilot cities, which can reduce repetitive and restrictive program strategies and improve the flexibility of policies in the implementation process. In addition, the second batch of pilot cities have got a clearer plan for the LTCI, which balances guidance and flexibility in policy design to adapt to local realities (64). Compared with the first batch of pilot cities, the second batch of pilot cities made a relatively high proportion of use of supply-based policies, with a significantly higher proportion of infrastructure construction, talent development and technology support. This indicates that the LTCI pilots are increasingly focusing on the construction of hard and soft facilities and the building of talent teams in care organizations, to ensure that material and human resources are sufficient. The use of demand-based policy tools in the first and second batches of pilot cities was low and did not show significant fluctuating changes, suggesting the need to further improve the vitality of the LTCI market (65).

### 4.2 Good overall rating for the LTCI policies

According to the analysis results of the PMC index model, the value of the PMC index for 79 LTCI policies was 7.701, with a policy rating of good. Among these, the policy ratings for the second batch of pilot cities were higher than those for the first batch. In terms of specific dimensions, the policies exhibited relatively short timeliness, lower policy levels, and room for improvement in incentive and constraint. However, it performed better in other dimensions such as policy objective and policy content. Furthermore, higher policy ratings corresponded with lower concavity in the PMC-surface. Detailed discussions and analyses of the policy ratings are presented below.

The values in terms of policy timeliness, policy level, and incentive and constraint were relatively low. LTCI policies have been in the pilot phase, and the timeliness of each policy is mainly short-term (1–3 years) and medium-term (3–5 years). At the same time, these policies can generally be used as normative documents and industry regulations, with a lack of more regulatory documents such as local laws and legislations, reducing the authority of the policies to a certain extent. Compared with countries such as Germany and Japan, which have already established corresponding legal systems for LTCI, the legislative and regulatory support system for LTCI in China is still relatively weak (66). This indicates that China lacks a unified top-level design for its LTCI policy, with significant variations among policy pilots in different areas, which is detrimental to the future unified management of the LTCI system. In addition, LTCI policies were currently weak in terms of incentive and constraint indicators, especially in terms of tax incentives, financial support, and government purchasing, which is not conducive to strengthening the supply of resources for the policies, promoting multi-party financing cooperation, and enhancing the market vitality on the demand side (53). Xue et al. conducted a PMC index model analysis on 36 LTCI policies issued at the central and provincial levels between 2016 and 2022. Their findings similarly rated the overall policy as good, while also identifying key developmental challenges as the lack of legal frameworks, insufficient effectiveness of incentive policy measures, and constraints on diversified funding channels (67).

LTCI policies had relatively high ratings in terms of policy nature, policy subject, policy objective, policy content, policy function, and policy evaluation, with mean values above 0.8. This can reflect the following aspects: first, LTCI policies have a better guiding role in prediction, recommendation, supervision, and description, and the policy objectives are clear, which helps to achieve the objectives of care guarantee for the disabled in the long-term, financial compensation, and the pilot extension of the policy (68), which was consistent with the findings of Xue et al. (67).

Second, LTCI policies cover a wide range of aspects such as financing, disability assessment, service provision, and treatment payment. The sustainable development of the LTCI depends on the policy design of the financing, assessment, safety provision and treatment segments, but the problems of a single financing channel and limited level of treatment need to be paid attention to (69). Wang et al. also pointed out in his research that broadening funding sources would enhance the fairness of LTCI financing (61).

Thirdly, the policy subjects involve the government, insurance companies, professional care organizations, social organizations, third-party assessment organizations, and the public, but the mechanism for deep cooperation and interaction among the subjects is still lacking. In addition, urban employees and urban and rural residents, as the general public, are to be fully included in the scope of LTCI coverage, but further studies have found that the coverage rate in rural areas in the pilot areas is relatively low, and the problem of imbalance between urban and rural areas in the development of LTCI is becoming increasingly prominent (70). Weng et al. noted in their research that ﻿older adults individuals in rural areas have faced poorer economic circumstances, lacked formal occupational safeguards, and received inadequate support from traditional family care models, with their carers themselves confronting greater challenges and pressures to sacrifice employment opportunities in order to provide care (63). The demand for LTC among the disabled ﻿older adults in rural areas is growing at a significantly higher rate than in urban areas. However, the majority of existing rehabilitation, medical, and healthcare facilities in China are concentrated in urban areas. This imbalance between supply and demand, coupled with the uneven distribution of resources, results in a poorer quality of life for the ﻿older adults with disability in rural areas, thereby further widening the wealth gap between urban and rural areas.

Fourthly, policies have positive functional value in terms of macro design, normative guidance, and service optimization. Taken together, the policies are formulated on well-founded, clear objectives and scientific programs, but there are relatively few measures to encourage incentives, supervision and constraints, so it is necessary to further improve the detailed nature of the policies (64). Li et al. also highlighted the need to further refine the LTC security and service system, detail policy development, and enhance the standard and quality of care services (8).

In terms of the phases, the mean of the PMC index of LTCI policies was highest in the second batch of pilot cities, second highest in the first batch of pilot cities in the first phase, and lowest in the first batch of pilot cities in the second phase. Regarding specific variables, the policies of the first batch of pilot cities in the first phase scored lower than the mean value, mainly in terms of the policy nature and function. This is related to the fact that LTCI policies did not significantly orient toward policy function in the early pilot phase. LTCI policies need to further clarify the policy nature and function in the course of practice, considering the care needs of the ﻿older adults and the accessibility of LTC services (71). The policies of the first batch of pilot cities in the second phase, as complementary policies to those of the first phase, scored relatively short in terms of timeliness, relatively low in terms of policy level, weak clarity in terms of policy objective, and left room for improvement in terms of incentive and constraint mechanisms and policy function. The second batch of pilot cities actively drew on the policy experience of the first batch of pilot cities, and improved their scores in terms of the policy nature, policy objective, and policy function (72). At the same time, the target of various policy measures has been made clearer, and the scope of policy subjects has been reduced accordingly, but the degree of closeness has been increased. Wang et al. also found in their research that compared to the first batch of pilot cities, the second batch of pilot cities saw a reduction in the scope of population coverage (61).

In addition, in terms of the PMC-surface of each rating policy, the PMC-surface of the perfect-rated policy was the least concave, and the values of the variables were relatively balanced. Hu et al. in their PMC index model analysis of ﻿older adults-related policies also noted that policies of higher rating were less prone to concavity (41). This has demonstrated that the perfect-rated policy has detailed provisions in all aspects of LTCI, such as policy targets, incentives and constraints, with comprehensive and substantive content, clear objectives and tasks, a well-defined functional nature, and few shortcomings, thereby effectively promoting the improvement of the LTCI. The PMC-surface of the poor-rated policy was the most concave, the values of the variables were at a low level, which can be blamed on the fact that the poor-rated policy is mainly a supplementary document for pilot projects and lacks substantive specific content. The degrees of PMC-surface concavity for the good- and acceptable-rated policies were in the middle of the above two, with a higher concavity in terms of policy timeliness, policy level, and incentive constraint variables compared with the perfect-rated policy. The concavity of the good-rated policy was lower than that of the acceptable-rated policy, and its concavity in terms of policy nature and policy function variables was lower than that of the acceptable-rated policy.

### 4.3 Differences in the choice of policy tools between different ratings of policies

The overall analysis showed that higher-rated policies employed a more balanced use of the various policy tools. The specific analysis is as follows.

The results of the analysis showed that perfect-rated policies had relatively weak reliance on environment-based policy tools and a relatively high degree of evenness in the use of all types of policy tools, followed by good-rated policies. Acceptable-rated policies were less even for using policy tools than good-rated policies, and poor-rated policies were the least even for using policy tools. This suggests that the ratings of LTCI policies are correlated with the evenness of using the policy tools. The ratings of LTCI policies can be improved by optimizing the use of various policy tools. In addition, compared to the first batch of pilot cities, the second batch of pilot cities had relatively higher evenness in using policy tools for each rating policy. This suggests that LTCI policies are continuous and evolving, and that the second batch of pilot cities have actively learned from the lessons of the first batch of pilot cities, further optimizing the use of policy tools and improving policy ratings.

### 4.4 Implications for practice

Based on the analysis of policy tools, although the second batch of pilot cities for LTCI demonstrated reduced reliance on environment-based policy tools, an overview of LTCI policies across all phases showed a continued preference for employing environment-based policy tools. In particular, the excessive use of three sub-policy tools, including strategic measures, institution building, and target planning, can lead to policies being more subject to traditional command-and-control administrative intervention during implementation, lacking flexibility for adjustment, and the implementation of policy content becoming mechanized and hollow. Consequently, the vital interests of the disabled ﻿older adults are not effectively protected. Conversely, the underutilized supply-based and demand-based policy tools has resulted in insufficient policy push and pull effects, limiting LTCI funding channels. This has also led to inadequate provision of hardware and software facilities alongside care professionals, insufficient activation of the demand-side market, and failure to fully meet the diverse, multi-tiered LTC needs of the disabled ﻿older adults. Therefore, the three types of policy tools should be used rationally, and the structure of sub-policy tools should be optimized. The specific recommendations are as follows.

Regarding environment-based policy tools, more attention should be paid to the effectiveness of three sub-policy tools, namely strategic measures, institution building, and target planning, while optimizing and upgrading those already overused to reduce the deployment of duplicative or ineffective policy tools. Meanwhile, efforts should be made to continuously explore and better utilize sub-policy tools such as policy publicity and tax incentives. For instance, publicity could be actively employed to raise awareness among disabled ﻿older adults individuals and their families about LTCI and encourage their acceptance of LTC services. For stakeholders involved in LTCI, preferential measures such as tax relief could be offered to attract greater social capital participation in related services. Through the rational deployment of policy tools, the government can both foster a conducive environment for the development of LTCI and promote its flexible, efficient implementation, thereby reducing reliance on traditional administrative intervention.

In response to the insufficient use of LTCI policies in supply-based policy tools, it is recommended that the government increase infrastructure investment, improve the training mechanism for LTC practitioners, enhance the support of informatization such as big data, and pay particular attention to the application of innovative internet technologies in LTCI management. It can be embedded in a standardized and scientific operation network through professional organizations, talents, capital and technology, to activate all kinds of resources and effectively promote the professional capacity of the supply of resources for LTC services.

To address the insufficient pull of demand-based policy tools, greater emphasis should be placed on utilizing such tools as government purchasing, market cultivation and policy subsidies. Market mechanisms should be introduced into the LTCI sector to fully leverage market and social forces, thereby fostering a socialized care services market capable of meeting diverse and multi-tiered LTC needs.

Analysis of the variables within the PMC index model has shown that the second phase of LTCI pilot cities has optimized policy nature and function. However, across all phases, LTCI policies predominantly take the form of normative documents and industry regulations, with relatively short policy timelines. This could lead to institutional fragmentation, potentially trapping LTCI development in short-termism while exacerbating regional and urban–rural disparities in coverage. The absence of incentive measures such as financial input, tax incentives, financial support, and talent training would lead to severe challenges for the sustainability of the LTCI fund, insufficient supply of professional care services. Consequently, public trust in LTCI would diminish, and the contradiction between increasing care demands of the disabled ﻿older adults and insufficient supply would persistently worsen. Specific recommendations are as follows.

Firstly, the central government should strengthen the top-level design of LTCI policies, make concerted efforts to establish a highly operational national implementation plan for LTCI, and expedite the issuance of a unified, reasonably funded, standardized, and integrated urban–rural national regulatory framework for LTCI. Local governments should continuously refine policy frameworks and implementation guidelines in accordance with central government policies. By prioritizing funding mechanisms and service optimization, they should vigorously develop the LTCI service market, continually improve the detailed design of the system, enhance policy effectiveness and enforceability, and standardize the operation of the system.

Secondly, it should leverage incentive policy measures to guide social forces, organizations and capital into the LTCI market in an orderly manner, building upon multi-channel funding sources, including individuals, government subsidies and charitable institutions, while offering multifaceted preferential policies and establishing incentive and constraint mechanisms to foster the sustainable development of LTCI. Concurrently, the government should increase investment in infrastructure, refine training mechanisms for LTC practitioners, and enhance support for information technology. This will fully leverage the critical role of “Internet Plus” and big data in LTCI services, enabling precision and intelligent care provision.

Furthermore, the government should expand the coverage of LTCI to gradually achieve comprehensive coverage across both urban and rural areas. On the one hand, the government should conduct policy publicity on LTCI in rural and remote areas, enabling more residents to understand, recognize and progressively participate in the system. On the other hand, the government should guide professional institutions to extend their services into rural and remote areas, and encourage more charitable organizations and local professionals in rural areas to engage in the LTCI sector, thereby providing increased care resources for the disabled ﻿older adults in these areas.

In summary, the government should optimize and upgrade policies that have been overused, and continuously explore and make use of more policy tools. Meanwhile, the LTCI policy can gradually extend its effective time and improve its authority. The incentive and constraint elements should also be strengthened to increase the supply of supporting policy resources, and to motivate the potential demand for LTCI among the service targets. In addition, the ratings of the policy can be improved by optimizing the use of policy tools and enhancing the guidance of the policy on the development of LTCI.

### 4.5 Limitations and future research

Although this study has its advantages, it also has some limitations. Firstly, there are various ways of classifying policy tools, and in this study, the supply-, environment-, and demand-based classification has been selected according to the content characteristics of the policy text. In future research, we can attempt to analyze the use of policy tools more comprehensively by choosing other ways of classifying policy tools, such as authoritative tools, capability-based tools, and learning tools. Second, the PMC index model has set the same weight for each evaluation variable. Although the refined and quantified difference in the setting of the second-level variables can feed the weights into the first-level variables to a certain extent, it does not reflect the weighting information in a complete and refined way. This consequently limits its effectiveness in identifying relationships between different evaluation indicators and significant relative differences. In future research, we will consider using methods such as the Analytic Hierarchy Process (AHP) to assign weights to the indicators at all levels, so as to evaluate the policy more objectively by reflecting the relative importance of each variable.

## 5 Conclusion

The choice of policy tools by the government had some preference, with over-utilization of environment-based policy tools and under-utilization of supply- and demand-based policy tools. The current LTCI policies have focused on macro-level environmental shaping, such as target planning and institutional development, but have been insufficient in guiding and activating both the supply and demand sides of the LTC service market. This can lead to vague policy content and inadequate implementation, thereby suppressing the supply of LTC services and market vitality, which can be attributed to the fact that the LTCI policy is still in the pilot phase. In the early stages, the policy relies on environment-based policy tools to provide a clear institutional framework and development environment. In the second batch of pilot cities, LTCI policies have become less reliant on environment-based policy tools. Moreover, the internal structure of each policy tool was unevenly distributed, which was more obvious in the policies of the first batch of pilot cities in the second phase, and it is necessary to rationally use the policy tools and optimize the internal structure.

The overall rating of LTCI policies in pilot cities was good. Among these, the second batch of pilot cities had higher policy ratings than those in the first batch. Overall, LTCI policies have been generally established and contribute to the development and improvement of the LTCI system. However, the timeliness of current policies still needs to be extended, the supporting legal and regulatory framework remains incomplete, and the policies lack incentives in areas such as taxation and finance. Additionally, the single-channel funding structure and limited coverage scope of LTCI also suggest that future efforts should focus on further exploring policy effectiveness, sustainable funding, and enhanced coverage to build a high-quality LTCI system. Combining the policy tools and policy ratings, policies of perfect, good, acceptable, and poor ratings showed that the evenness of using policy tools decreases in turn. In addition, compared to the first batch of pilot cities, the second batch of pilot cities had more even in the use of policy tools for each rating policy.

## Data Availability

The original contributions presented in the study are included in the article/Supplementary material, further inquiries can be directed to the corresponding author.
