# Supplementary material for: How to evaluate long-term care insurance policy based on policy tools and PMC index model: evidence from pilot cities in China
Source: Front Public Health. 2025 Oct 6;13:1661785. doi: 10.3389/fpubh.2025.1661785 (PMC12536656; doi:10.3389/fpubh.2025.1661785)
Supplement: Supplementary file 1 [file Supplementary_file_1.docx]

Table S1 Summary of the LTCI policy in China

| Policy | Main objectives | Main content | Department of issue | Year of issue |
| --- | --- | --- | --- | --- |
| Guidance opinions on launching the pilot long-term care insurance system | (a) Using a pilot period of one to two years, to basically form a policy framework for a LTCI system that is adapted to Chinese conditions  (b) Exploring the policy systems and operating mechanisms of LTCI, such as the scope of coverage, financing and payment, requirements rating assessment, quality assessment, and standardized management | (a) Preliminary provisions on LTCI coverage and protection, fund-raising and treatment payment, management of the fund and handling services, corresponding supporting measures, and organization and implementation  (b) Identifying 15 pilot cities for LTCI and two key contact provinces | Ministry of Human Resources and Social Security | 2016 |
| Guiding opinions on expanding the pilot long-term care insurance system | Establishing a social insurance system with service or funding coverage for long-term disability, basically forming a policy framework for the LTCI system adapted to Chinese conditions, and establishing and improving a multi-level LTCI system that met the multiple demands of the public | (a) Further clarifying LTCI participation and coverage, fund-raising and treatment payment, management of funds and handling services, and organization and implementation.  (b) Adding 14 new pilot cities for LTCI | National Healthcare Security Administration,  Ministry of Finance | 2020 |
| Criteria for assessing the disability level of long-term care (trial implementation) | (a) Using to guide the LTCI disability rating assessment conducted by the healthcare security departments in the pilot cities of the LTCI system  (b) Providing references to the Ministry of Civil Affairs with eligibility determination for elderly care subsidies and evaluation of admission assessment to elderly care organizations in the pilot cities of the LTCI system | (a) Identifying the subject, object, place, process, and requirements for disability assessment  (b) Provision of disability assessment scores and ratings | National Healthcare Security Administration, Ministry of Civil Affairs | 2021 |
| Administrative measures for assessing the disability level of long-term care insurance (trial implementation) | Strengthening the management of LTCI disability level assessment, as an important basis for the LTCI fund to pay for treatment and formulate handling procedures | Regulating requirements for the designated organizations, personnel, process standards, supervision, and management of the disability-level assessment | National Healthcare Security Administration,  Ministry of Finance | 2023 |
| Designated administrative measures for long-term care insurance disability-level assessment organizations (trial run) | Preparation of a model service agreement to guide localities in the provision of designated management services for assessment organizations | Clarifying the criteria for determining the designated assessment organizations, operation and management, supervision and management, and other related matters | National Healthcare Security Administration | 2024 |
| Designated administrative measures for care service organizations of long-term care insurance (trial implementation) | For management of designated LTC service organizations in areas where the LTCI system is implemented | Clarifying matters relating to the determination of designated LTC service organizations, operation and management, management of services, dynamic management, supervision and management, and so on | National Healthcare Security Administration | 2024 |
| Handling procedures of long-term care insurance (trial implementation) | Ensuring the orderly operation of LTCI, building an operational system based on government running and supplemented by social forces, and providing convenient and efficient handling services | Clarifying matters such as assessment of disability, management of agreements with assessment organizations, care services, management of agreements with LTC service organizations, management of funds, auditing and verification, information technology and file management, and social participation | National Healthcare Security Administration | 2024 |

Table S2 Text of long-term care insurance policies for the first and second batches of pilot cities

| Policy number | Policy Name | Department of issue | Time of issue |
| --- | --- | --- | --- |
| 1 | Implementation Opinions of Chengde People's Government on Establishing Long-term Care Insurance System for Urban Employees | Chengde Municipal Government | November 2016 |
| 2 | Implementation Plan for Long-term Care Insurance in Qiqihar City (Trial) | Qiqihar Municipal Government | July 2017 |
| 3 | Implementation Rules for Long-term Care Insurance in Qiqihar City (Trial) | Qiqihar Municipal Human Resources and Social Security Bureau | September 2017 |
| 4 | Pilot Measures for Long-term Care Insurance in Shanghai | [Shanghai Municipal Government](http://www.pkulaw.cn/cluster_form.aspx?Db=lar&EncodingName=&search_tj=fdep_id%7b3a80902) | December 2016 |
| 5 | Implementation Rules for the Pilot Measures of Long-term Care Insurance in Shanghai (Trial) | Shanghai Municipal Human Resources and Social Security Bureau, Shanghai Municipal Healthcare Insurance Office | December 2016 |
| 6 | Pilot Measures for Long-term Care Insurance in Shanghai | Shanghai Municipal Government | December 2017 |
| 7 | Implementation Rules for the Pilot Measures of Long-term Care Insurance in Shanghai (Trial) | Shanghai Municipal Human Resources and Social Security Bureau, Shanghai Municipal Healthcare Insurance Office | December 2017 |
| 8 | Implementation Rules for the Pilot Measures of Long-term Care Insurance in Shanghai (Trial) | Shanghai Municipal Healthcare Security Bureau | December 2019 |
| 9 | Implementation Rules for Basic Care Insurance in Nantong City | Nantong Municipal Human Resources and Social Security Bureau | December 2016 |
| 10 | Notice on Improving the Relevant Regulations of Long-term Care Insurance | Nantong Municipal Healthcare Security Bureau | August  2019 |
| 11 | Notice on Improving the Relevant Regulations of Long-term Care Insurance | Nantong Municipal Healthcare Security Bureau | March  2020 |
| 12 | Notice on the Implementation of Long-term Care Insurance Pilot Program | Suzhou Municipal Government | September 2017 |
| 13 | Implementation Opinions on Carrying out the Second Phase of Long-term Care Insurance Pilot Work | Suzhou Municipal Government | August  2020 |
| 14 | Pilot Plan for Long-term Care Insurance System in Ningbo City | Ningbo Municipal Government | September 2017 |
| 15 | Implementation Rules for Long-term Care Insurance Pilot in Ningbo City | Ningbo Municipal Human Resources and Social Security Bureau, Ningbo Municipal Finance Bureau, Ningbo Municipal Civil Affairs Bureau | December 2017 |
| 16 | Implementation Opinions on the Pilot Program of Long-term Care Insurance for Urban Employees in Anqing City | Anqing Municipal Government | January 2017 |
| 17 | Implementation Rules for Long-term Care Insurance for Employees in Anqing City (Trial) | Anqing Municipal Human Resources and Social Security Bureau, Anqing Municipal Finance Bureau | April  2017 |
| 18 | Implementation Measures for Long-term Care Insurance for Urban Employees in Anqing City | Anqing Municipal Government | January 2020 |
| 19 | Implementation Plan for Launching Pilot Work of Long-term Care Insurance | Shangrao Municipal Government | December 2016 |
| 20 | Implementation Plan for Comprehensive Pilot of Long-term Care Insurance System | Shangrao Municipal Government | July  2019 |
| 21 | Interim Measures for Long-term Care Insurance in Qingdao City | Qingdao Municipal Government | February 2018 |
| 22 | Measures for Long-term Care Insurance in Qingdao City | Qingdao Municipal Government | March 2020 |
| 23 | Measures for Long-term Care Insurance in Jingmen City (Trial) | Jingmen Municipal Government | November 2016 |
| 24 | Implementation Rules for Long-term Care Insurance in Jingmen City (Trial), Management Measures for Designated Service Organizations of Long-term Care Insurance in Jingmen City (Trial) | Jingmen Municipal Human Resources and Social Security Bureau | December 2016 |
| 25 | Trial Measures for Long-term Care Insurance in Guangzhou | Guangzhou Municipal Human Resources and Social Security Bureau, Guangzhou Municipal Finance Bureau, Guangzhou Municipal Civil Affairs Bureau, Guangzhou Municipal Health Commission | July  2017 |
| 26 | Trial Measures for Long-term Care Insurance in Guangzhou | Guangzhou Municipal Healthcare Security Bureau, Guangzhou Municipal Finance Bureau, Guangzhou Municipal Civil Affairs Bureau, Guangzhou Municipal Health Commission | July  2019 |
| 27 | Opinions on the Pilot Program of Long-term Care Insurance System in Chongqing | Chongqing Municipal Human Resources and Social Security Bureau, Chongqing Municipal Finance Bureau | December 2017 |
| 28 | Implementation Rules for Long-term Care Insurance in Chongqing (Trial) | Chongqing Municipal Healthcare Security Bureau | December 2018 |
| 29 | Pilot Plan for Long-term Care Insurance System in Chengdu City | Chengdu Municipal Government | February 2017 |
| 30 | Implementation Rules for Long-term Care Insurance in Chengdu (Trial) | Chengdu Municipal Human Resources and Social Security Bureau, Chengdu Municipal Finance Bureau | April  2017 |
| 31 | Implementation Opinions of Chengdu Municipal People's Government on Deepening the Pilot of Long-term Care Insurance System | Chengdu Municipal Government | May  2020 |
| 32 | Implementation Rules for Long-term Care Insurance for Urban Employees in Chengdu City | Chengdu Municipal Healthcare Security Bureau, Chengdu Municipal Finance Bureau, Chengdu Municipal Human Resources and Social Security Bureau, Chengdu Municipal Tax Service Administration | May  2020 |
| 33 | Implementation Rules for Long-term Care Insurance for Urban and Rural Residents in Chengdu | Chengdu Municipal Healthcare Security Bureau, Chengdu Municipal Finance Bureau, Chengdu Municipal Human Resources and Social Security Bureau, Chengdu Municipal Tax Service Administration | May  2020 |
| 34 | Opinions on Establishing a Long-term Care Insurance System (Trial) | Office of the Eighth Division of the Xinjiang Production and Construction Corps, Shihezi Municipal Government | March  2017 |
| 35 | Implementation Rules for Long-term Care Insurance in Shihezi City, Eighth Division (Trial) | Office of the Eighth Division of the Xinjiang Production and Construction Corps, Shihezi Municipal Government | March  2017 |
| 36 | Guiding Opinions on Expanding the Pilot Program of Long-term Care Insurance System | Hebei Provincial Healthcare Security Bureau, Hebei Provincial Department of Finance | March  2021 |
| 37 | Management Measures for Long-term Care Insurance for Urban Employees in Chengde City | Chengde Municipal Healthcare Security Bureau, Chengde Municipal Finance Bureau | June  2021 |
| 38 | Notice of Changchun Medical Security Bureau on Expanding the Pilot Work of Medical Care Insurance System for Disabled Persons | Changchun Municipal Healthcare Security Bureau | December 2021 |
| 39 | Pilot Implementation Plan for Deepening Long-term Care Insurance System in Qiqihar City (Trial) | Qiqihar Municipal Government | February 2021 |
| 40 | Pilot Measures for Long-term Care Insurance in Shanghai | Shanghai Municipal Government | December 2021 |
| 41 | Implementation Opinions on Further Promoting the Pilot Work of Long-term Care Insurance | Suzhou Municipal Government | October 2022 |
| 42 | Guiding Opinions of the General Office of the People's Government of Ningbo Municipality on Deepening the Pilot of Long-term Care Insurance System | Ningbo Municipal Government | August  2022 |
| 43 | Notice on Adjusting the Policies Related to Long-term Care Insurance for Urban Employees in Anqing City | Anqing Municipal Healthcare Security Bureau, Anqing Municipal Finance Bureau | October 2021 |
| 44 | Measures for Long-term Care Insurance in Qingdao City | Qingdao Municipal Government | March 2021 |
| 45 | Trial Measures for Long-term Care Insurance in Guangzhou | Guangzhou Municipal Healthcare Security Bureau, Guangzhou Municipal Finance Bureau, Guangzhou Municipal Civil Affairs Bureau, Guangzhou Municipal Health Commission | December 2020 |
| 46 | Trial Measures for Long-term Care Insurance in Guangzhou | Guangzhou Municipal Healthcare Security Bureau, Guangzhou Municipal Finance Bureau, Guangzhou Municipal Civil Affairs Bureau, Guangzhou Municipal Health Commission | January 2024 |
| 47 | Implementation Opinions of Chongqing Medical Security Bureau and Chongqing Finance Bureau on Expanding the Pilot of Long-term Care Insurance System | Chongqing Municipal Healthcare Security Bureau, Chongqing Municipal Finance Bureau | November 2021 |
| 48 | Implementation Opinions of Chengdu Municipal People's Government on Launching a New Round of Long-term Care Insurance Reform | Chengdu Municipal Government | May 2022 |
| 49 | Implementation Rules for Long-term Care Insurance in Chengdu City | Chengdu Municipal Healthcare Security Bureau, Chengdu Municipal Civil Affairs Bureau, Chengdu Municipal Finance Bureau, Chengdu Municipal Human Resources and Social Security Bureau, Chengdu Health Commission, Chengdu Municipal Tax Administration | June 2022 |
| 50 | Implementation Plan for Pilot Expansion of Long-term Care Insurance System in Shijingshan District, Beijing | Shijingshan District Healthcare Security Bureau and Shijingshan District Finance Bureau of Beijing Municipality | November 2020 |
| 51 | Implementation Rules for the Pilot Project of Expanding Long-term Care Insurance System in Shijingshan District, Beijing | Shijingshan District Healthcare Security Bureau and Shijingshan District Finance Bureau of Beijing Municipality | November 2020 |
| 52 | Pilot Implementation Plan for Long-term Care Insurance System in Tianjin | Tianjin Municipal Government | December 2020 |
| 53 | Implementation Rules for the Pilot Implementation Plan of Long-term Care Insurance System in Tianjin (Trial) | Tianjin Municipal Healthcare Security Bureau | January 2021 |
| 54 | Implementation Plan for Deepening the Pilot Program of Long-term Care Insurance System in Tianjin | Tianjin Municipal Government | December 2022 |
| 55 | Implementation Details of the Implementation Plan for Deepening the Pilot Program of Long-term Care Insurance System | Tianjin Municipal Healthcare Insurance Bureau, Tianjin Municipal Finance Bureau, Tianjin Municipal Civil Affairs Bureau, Tianjin Municipal Human Resources and Social Security Bureau, Tianjin Municipal Health Commission, Tianjin Municipal Tax Administration, Tianjin Banking and Insurance Regulatory Bureau | December 2022 |
| 56 | Implementation Opinions on Establishing a Long-term Care Insurance System | Jincheng Municipal Government | November 2020 |
| 57 | Implementation Rules for Long-term Care Insurance for Employees in Jincheng City | Jincheng Municipal Healthcare Security Bureau, Jincheng Municipal Civil Affairs Bureau, Jincheng Municipal Finance Bureau, Jincheng Municipal Human Resources and Social Security Bureau, Jincheng Municipal Health Commission | February 2021 |
| 58 | Pilot Implementation Plan for Long-term Care Insurance System in Hohhot City | Hohhot Municipal Government | December 2020 |
| 59 | Implementation Plan for Pilot Work of National Long-term Care Insurance System in Panjin City | Panjin Municipal Government | December 2020 |
| 60 | Implementation Plan for Pilot Long-term Care Insurance System | Fuzhou Municipal Government | December 2020 |
| 61 | Implementation Rules for Long-term Care Insurance in Fuzhou City | Fuzhou Municipal Healthcare Security Bureau, Fuzhou Municipal Finance Bureau, Fuzhou Municipal Health Commission, Fuzhou Municipal Civil Affairs Bureau, Fuzhou Municipal Tax Administration | March  2021 |
| 62 | Trial Measures for Long-term Care Insurance System in Kaifeng City | Kaifeng Municipal Government | December 2020 |
| 63 | Trial Measures for Long-term Care Insurance System in Kaifeng City | Kaifeng Municipal Government | July 2021 |
| 64 | Pilot Implementation Plan for Long-term Care Insurance System in Xiangtan City | Xiangtan Municipal Government | December 2020 |
| 65 | Implementation Rules for Long-term Care Insurance System in Xiangtan City (Trial) | Xiangtan Municipal Healthcare Security Bureau | January 2021 |
| 66 | Implementation Opinions of Nanning Municipal People's Government on the Pilot of Long-term Care Insurance System in Nanning City | Nanning Municipal Government | January 2021 |
| 67 | Implementation Measures for the Pilot of Long-term Care Insurance System in Nanning City | Nanning Municipal Healthcare Security Bureau, Nanning Municipal Finance Bureau, Nanning Municipal Human Resources and Social Security Bureau, Nanning Municipal Health Commission, Nanning Municipal Civil Affairs Bureau, Nanning Municipal Tax Administration, Nanning Municipal Disabled Persons' Federation | January 2021 |
| 68 | Implementation Opinions of Nanning Municipal People's Government on Promoting the Long-term Care Insurance System in Nanning City | Nanning Municipal Government | January 2024 |
| 69 | Pilot Implementation Plan for Long-term Care Insurance System in Qiannan Prefecture | Qiannan Prefecture Municipal Government | November 2020 |
| 70 | Trial Implementation Rules for Long-term Care Insurance in Qiannan Prefecture | Qiannan Prefecture Healthcare Security Bureau, Qiannan Prefecture Finance Bureau | December 2020 |
| 71 | Revised Implementation Plan for Long-term Care Insurance System Pilot in Qiannan Prefecture | Qiannan Prefecture Municipal Government | October 2022 |
| 72 | Comprehensive Pilot Work Plan for Long-term Care Insurance System | Kunming Municipal Government | December 2020 |
| 73 | Comprehensive Pilot Work Plan for Long-term Care Insurance System (Revised Edition) | Kunming Municipal Government | February 2023 |
| 74 | Implementation Measures for Long-term Care Insurance in Hanzhong City (Trial) | Hanzhong Municipal Government | November 2020 |
| 75 | Implementation Rules for Long-term Care Insurance in Hanzhong City (Trial) | Hanzhong Municipal Healthcare Insurance Bureau | April 2021 |
| 76 | Implementation Rules for Long-term Care Insurance for Employees in Gannan Prefecture (Trial) | Gannan Prefecture Healthcare Security Bureau, Gannan Prefecture Finance Bureau | November 2021 |
| 77 | Notice on the Pilot Implementation of Long-term Care Insurance System in Urumqi City | Autonomous Region Medical Security Bureau | November 2020 |
| 78 | Urumqi Long-term Nursing Insurance Measures | Urumqi Municipal Government | November 2021 |
| 79 | Implementation Rules for Long-term Care Insurance in Urumqi City | Urumqi Municipal Healthcare Security Bureau | March 2022 |

Table S3 Results of the PMC index for Long-term care insurance policies in China

| Policy number | X_1_ | X_2_ | X_3_ | X_4_ | X_5_ | X_6_ | X_7_ | X_8_ | X_9_ | X_10_ | PMC index | Ranking | Rating |
| --- | --- | --- | --- | --- | --- | --- | --- | --- | --- | --- | --- | --- | --- |
| 1 | 0.833 | 0.250 | 0.750 | 0.857 | 1 | 1 | 0.857 | 1 | 1 | 1 | 8.547 | 12 | good |
| 2 | 0.833 | 0.250 | 0.500 | 0.857 | 1 | 0.833 | 0.714 | 1 | 1 | 1 | 7.987 | 40 | good |
| 3 | 0.833 | 0.250 | 0.500 | 0.857 | 1 | 1 | 0.286 | 1 | 1 | 1 | 7.726 | 51 | good |
| 4 | 1 | 0.250 | 0.750 | 1 | 1 | 1 | 0.714 | 1 | 1 | 1 | 8.714 | 4 | good |
| 5 | 0.500 | 0.250 | 0.500 | 0.571 | 1 | 1 | 0.143 | 0.400 | 1 | 1 | 6.364 | 72 | acceptable |
| 6 | 1 | 0.250 | 0.750 | 1 | 1 | 1 | 0.714 | 1 | 1 | 1 | 8.714 | 4 | good |
| 7 | 0.500 | 0.250 | 0.500 | 0.714 | 1 | 1 | 0.143 | 0.400 | 1 | 1 | 6.507 | 69 | acceptable |
| 8 | 0.500 | 0.250 | 0.500 | 0.714 | 1 | 1 | 0.143 | 0.400 | 1 | 1 | 6.507 | 69 | acceptable |
| 9 | 0.667 | 0.250 | 0.500 | 0.571 | 1 | 1 | 0.286 | 0.800 | 0.750 | 1 | 6.824 | 62 | acceptable |
| 10 | 0.500 | 0.250 | 0.500 | 0.571 | 1 | 0.667 | 0.143 | 0.400 | 0.750 | 1 | 5.781 | 77 | acceptable |
| 11 | 0.500 | 0.250 | 0.500 | 0.571 | 0.667 | 0.833 | 0.429 | 0.600 | 0.750 | 1 | 6.100 | 75 | acceptable |
| 12 | 1 | 0.250 | 0.750 | 1 | 1 | 1 | 0.714 | 1 | 1 | 1 | 8.714 | 4 | good |
| 13 | 1 | 0.250 | 0.750 | 0.857 | 1 | 1 | 0.429 | 1 | 1 | 1 | 8.286 | 23 | good |
| 14 | 1 | 0.500 | 0.500 | 1 | 1 | 1 | 1 | 1 | 1 | 1 | 9.000 | 2 | perfect |
| 15 | 0.500 | 0.250 | 0.500 | 1 | 0.667 | 1 | 0.429 | 0.800 | 0.750 | 1 | 6.896 | 60 | acceptable |
| 16 | 1 | 0.500 | 0.500 | 1 | 1 | 1 | 1 | 1 | 1 | 1 | 9.000 | 2 | perfect |
| 17 | 0.500 | 0.250 | 0.500 | 0.857 | 1 | 0.667 | 0.286 | 0.400 | 0.500 | 1 | 5.960 | 76 | acceptable |
| 18 | 0.500 | 0.250 | 0.500 | 0.857 | 1 | 1 | 0.429 | 0.400 | 0.500 | 1 | 6.436 | 71 | acceptable |
| 19 | 0.833 | 0.500 | 0.500 | 0.857 | 1 | 0.833 | 1 | 1 | 1 | 1 | 8.523 | 15 | good |
| 20 | 1 | 0.500 | 0.750 | 1 | 1 | 1 | 0.857 | 1 | 1 | 1 | 9.107 | 1 | perfect |
| 21 | 0.833 | 0.250 | 0.750 | 0.857 | 1 | 1 | 0.714 | 1 | 1 | 1 | 8.404 | 19 | good |
| 22 | 0.833 | 0.250 | 0.750 | 0.857 | 1 | 1 | 0.714 | 1 | 1 | 1 | 8.404 | 19 | good |
| 23 | 0.833 | 0.500 | 0.750 | 0.714 | 1 | 1 | 0.714 | 1 | 1 | 1 | 8.511 | 16 | good |
| 24 | 0.833 | 0.500 | 0.500 | 0.714 | 1 | 1 | 1 | 1 | 1 | 1 | 8.547 | 12 | good |
| 25 | 1 | 0.250 | 0.500 | 0.857 | 1 | 0.833 | 0.571 | 0.800 | 0.750 | 1 | 7.561 | 56 | good |
| 26 | 0.833 | 0.250 | 0.500 | 0.714 | 0.667 | 0.667 | 0.286 | 0.600 | 0.750 | 1 | 6.267 | 74 | acceptable |
| 27 | 0.833 | 0.500 | 0.500 | 0.714 | 1 | 0.667 | 0.857 | 1 | 0.800 | 1 | 7.871 | 45 | good |
| 28 | 0.667 | 0.250 | 0.500 | 0.714 | 0.333 | 0.667 | 0.571 | 0.800 | 0.800 | 1 | 6.302 | 73 | acceptable |
| 29 | 1 | 0.250 | 0.750 | 0.571 | 1 | 1 | 0.714 | 1 | 1 | 1 | 8.285 | 25 | good |
| 30 | 0.833 | 0.250 | 0.500 | 1 | 0.667 | 1 | 0.714 | 0.800 | 1 | 1 | 7.764 | 50 | good |
| 31 | 1 | 0.250 | 0.500 | 1 | 1 | 1 | 0.429 | 1 | 1 | 1 | 8.179 | 28 | good |
| 32 | 0.667 | 0.250 | 0.500 | 1 | 1 | 1 | 0.714 | 0.800 | 1 | 1 | 7.931 | 42 | good |
| 33 | 1 | 0.250 | 0.500 | 0.714 | 0.667 | 0.833 | 0.571 | 0.800 | 1 | 1 | 7.335 | 57 | good |
| 34 | 0.833 | 0.250 | 0.750 | 1 | 1 | 0.667 | 0.714 | 1 | 0.750 | 1 | 7.964 | 41 | good |
| 35 | 0.833 | 0.250 | 0.500 | 1 | 1 | 1 | 0.571 | 1 | 0.750 | 1 | 7.904 | 43 | good |
| 36 | 0.833 | 0.500 | 0.500 | 0.571 | 1 | 0.833 | 0.143 | 0.600 | 0.750 | 1 | 6.730 | 67 | acceptable |
| 37 | 0.833 | 0.250 | 0.500 | 0.857 | 1 | 1 | 0.429 | 0.800 | 1 | 1 | 7.669 | 54 | good |
| 38 | 0.833 | 0.250 | 0.500 | 0.714 | 1 | 0.833 | 0.143 | 0.600 | 1 | 1 | 6.873 | 61 | acceptable |
| 39 | 1 | 0.250 | 0.500 | 0.857 | 1 | 1 | 1 | 1 | 1 | 1 | 8.607 | 7 | good |
| 40 | 1 | 0.250 | 0.500 | 1 | 1 | 1 | 0.714 | 1 | 1 | 1 | 8.464 | 17 | good |
| 41 | 1 | 0.250 | 0.750 | 0.857 | 1 | 1 | 0.571 | 1 | 1 | 1 | 8.428 | 18 | good |
| 42 | 1 | 0.250 | 0.500 | 0.857 | 1 | 1 | 0.571 | 1 | 1 | 1 | 8.178 | 29 | good |
| 43 | 0.333 | 0.250 | 0.250 | 0.429 | 0.667 | 0.667 | 0.143 | 0.200 | 0.500 | 1 | 4.439 | 79 | poor |
| 44 | 0.833 | 0.250 | 0.750 | 1 | 1 | 1 | 0.714 | 1 | 1 | 1 | 8.547 | 12 | good |
| 45 | 1 | 0.250 | 0.500 | 0.857 | 0.667 | 1 | 0.429 | 1 | 1 | 1 | 7.703 | 53 | good |
| 46 | 1 | 0.250 | 0.500 | 1 | 1 | 1 | 0.286 | 1 | 1 | 1 | 8.036 | 37 | good |
| 47 | 1 | 0.250 | 0.500 | 0.714 | 0.667 | 0.833 | 0.286 | 1 | 0.800 | 1 | 7.050 | 58 | good |
| 48 | 0.833 | 0.250 | 0.750 | 1 | 0.667 | 1 | 0.714 | 0.600 | 0.750 | 1 | 7.564 | 55 | good |
| 49 | 0.667 | 0.250 | 0.500 | 1 | 1 | 1 | 0.571 | 0.800 | 1 | 1 | 7.788 | 49 | good |
| 50 | 0.833 | 0.250 | 0.500 | 0.571 | 0.667 | 1 | 0.429 | 0.800 | 0.750 | 1 | 6.800 | 64 | acceptable |
| 51 | 1 | 0.500 | 0.500 | 1 | 1 | 1 | 0.571 | 1 | 1 | 1 | 8.571 | 8 | good |
| 52 | 0.833 | 0.250 | 0.500 | 0.857 | 1 | 1 | 0.571 | 1 | 1 | 1 | 8.011 | 39 | good |
| 53 | 1 | 0.250 | 0.500 | 0.714 | 1 | 1 | 0.429 | 1 | 1 | 1 | 7.893 | 44 | good |
| 54 | 1 | 0.250 | 0.500 | 0.714 | 1 | 1 | 0.571 | 1 | 1 | 1 | 8.035 | 38 | good |
| 55 | 0.667 | 0.250 | 0.500 | 0.571 | 1 | 0.667 | 0.429 | 0.800 | 0.750 | 1 | 6.634 | 68 | acceptable |
| 56 | 0.833 | 0.250 | 0.750 | 1 | 1 | 0.833 | 0.714 | 1 | 1 | 1 | 8.380 | 21 | good |
| 57 | 0.833 | 0.500 | 0.500 | 1 | 1 | 0.833 | 0.429 | 1 | 1 | 1 | 8.095 | 36 | good |
| 58 | 0.833 | 0.500 | 0.500 | 0.571 | 1 | 1 | 0.714 | 1 | 1 | 1 | 8.118 | 34 | good |
| 59 | 1 | 0.500 | 0.500 | 0.571 | 1 | 1 | 0.571 | 1 | 1 | 1 | 8.142 | 33 | good |
| 60 | 0.833 | 0.250 | 0.500 | 0.429 | 1 | 0.833 | 0.429 | 0.800 | 0.750 | 1 | 6.824 | 62 | acceptable |
| 61 | 1 | 0.500 | 0.500 | 1 | 1 | 1 | 0.571 | 1 | 1 | 1 | 8.571 | 8 | good |
| 62 | 1 | 0.250 | 0.750 | 0.714 | 1 | 1 | 0.571 | 1 | 1 | 1 | 8.285 | 25 | good |
| 63 | 1 | 0.250 | 0.750 | 0.714 | 1 | 1 | 0.571 | 1 | 1 | 1 | 8.285 | 25 | good |
| 64 | 0.833 | 0.250 | 0.500 | 0.714 | 1 | 1 | 0.571 | 1 | 1 | 1 | 7.868 | 47 | good |
| 65 | 1 | 0.250 | 0.500 | 0.857 | 1 | 1 | 0.571 | 1 | 1 | 1 | 8.178 | 29 | good |
| 66 | 1 | 0.250 | 0.750 | 0.714 | 1 | 1 | 0.429 | 1 | 1 | 1 | 8.143 | 32 | good |
| 67 | 0.714 | 0.250 | 0.500 | 0.714 | 1 | 1 | 0.429 | 0.600 | 0.750 | 1 | 6.957 | 59 | acceptable |
| 68 | 0.833 | 0.250 | 0.750 | 0.714 | 1 | 1 | 0.571 | 1 | 1 | 1 | 8.118 | 34 | good |
| 69 | 0.667 | 0.500 | 0.500 | 0.833 | 0.667 | 0.500 | 0.571 | 0.800 | 0.750 | 1 | 6.788 | 66 | acceptable |
| 70 | 0.833 | 0.500 | 0.500 | 0.714 | 1 | 1 | 0.286 | 1 | 1 | 1 | 7.833 | 48 | good |
| 71 | 0.833 | 0.250 | 0.500 | 0.714 | 1 | 0.667 | 0.286 | 0.800 | 0.750 | 1 | 6.800 | 64 | acceptable |
| 72 | 1 | 0.500 | 0.750 | 0.857 | 1 | 1 | 0.714 | 1 | 0.750 | 1 | 8.571 | 8 | good |
| 73 | 0.833 | 0.250 | 0.750 | 0.714 | 1 | 1 | 0.429 | 1 | 0.750 | 1 | 7.726 | 51 | good |
| 74 | 0.833 | 0.250 | 0.500 | 0.857 | 1 | 1 | 0.429 | 1 | 1 | 1 | 7.869 | 46 | good |
| 75 | 1 | 0.250 | 0.500 | 0.857 | 1 | 1 | 0.571 | 1 | 1 | 1 | 8.178 | 29 | good |
| 76 | 1 | 0.500 | 0.500 | 0.857 | 1 | 1 | 0.429 | 1 | 1 | 1 | 8.286 | 23 | good |
| 77 | 0.333 | 0.250 | 0.500 | 0.429 | 0.333 | 0.333 | 0.286 | 0.600 | 0.500 | 1 | 4.564 | 78 | poor |
| 78 | 1 | 0.250 | 0.500 | 1 | 1 | 1 | 0.571 | 1 | 1 | 1 | 8.321 | 22 | good |
| 79 | 1 | 0.500 | 0.500 | 1 | 1 | 1 | 0.571 | 1 | 1 | 1 | 8.571 | 8 | good |

Table S4 Two-dimensional distribution of the LTCI policy tools and policy ratings

| Policy tools | | Policies of the first batch of pilot cities in the first phase | | | |  | Policies of the first batch of pilot cities in the second phase | | | |  | Policies of the second batch of pilot cities | | | |
| --- | --- | --- | --- | --- | --- | --- | --- | --- | --- | --- | --- | --- | --- | --- | --- |
|  |  | perfect | good | acceptable | poor |  | perfect | good | acceptable | poor |  | perfect | good | acceptable | poor |
| Supply-based | Infrastructure construction | 2  (0.148) | 33  (2.437) | 13  (0.960) | 0  (0) |  | 0  (0) | 15  (3.043) | 0  (0) | 0  (0) |  | 0  (0) | 43  (4.030) | 11  (1.031) | 0  (0) |
|  | Technology support | 0  (0) | 0  (0) | 1  (0.074) | 0  (0) |  | 0  (0) | 0  (0) | 0  (0) | 0  (0) |  | 0  (0) | 5  (0.469) | 3  (0.281) | 0  (0) |
|  | Talent development | 4  (0.295) | 26  (1.920) | 7  (0.517) | 0  (0) |  | 0  (0) | 10  (2.028) | 0  (0) | 0  (0) |  | 0  (0) | 36  (3.374) | 8  (0.750) | 0  (0) |
|  | Information support | 5  (0.369) | 30  (2.216) | 1  (0.074) | 0  (0) |  | 0  (0) | 8  (1.623) | 0  (0) | 0  (0) |  | 0  (0) | 21  (1.968) | 2  (0.187) | 0  (0) |
|  | Financial support | 1  (0.074) | 41  (3.028) | 0  (0) | 0  (0) |  | 0  (0) | 18  (3.651) | 0  (0) | 0  (0) |  | 0  (0) | 36  (3.374) | 6  (0.562) | 0  (0) |
|  | Total | 12  (0.886) | 130  (9.601) | 22  (1.625) | 0  (0) |  | 0  (0) | 51  (10.345) | 0  (0) | 0  (0) |  | 0  (0) | 141  (13.215) | 30  (2.812) | 0  (0) |
| Environment-based | Tax  incentives | 1  (0.074) | 4  (0.295) | 0  (0) | 0  (0) |  | 0  (0) | 1  (0.203) | 0  (0) | 0  (0) |  | 0  (0) | 2  (0.187) | 0  (0) | 0  (0) |
|  | Strategic measures | 46  (3.397) | 535  (39.513) | 222  (16.396) | 0  (0) |  | 0  (0) | 275  (55.781) | 12  (2.434) | 2  (0.406) |  | 0  (0) | 499  (46.767) | 101  (9.466) | 1  (0.094) |
|  | Regulatory control | 4  (0.295) | 23  (1.699) | 8  (0.591) | 0  (0) |  | 0  (0) | 20  (4.057) | 0  (0) | 0  (0) |  | 0  (0) | 18  (1.687) | 4  (0.375) | 0  (0) |
|  | Institution building | 11  (0.812) | 76  (5.613) | 12  (0.886) | 0  (0) |  | 0  (0) | 32  (6.491) | 3  (0.609) | 0  (0) |  | 0  (0) | 38  (3.561) | 12  (1.125) | 2  (0.187) |
|  | Target planning | 9  (0.665) | 68  (5.022) | 22  (1.625) | 0  (0) |  | 0  (0) | 35  (7.099) | 3  (0.609) | 1  (0.203) |  | 0  (0) | 73  (6.842) | 16  (1.500) | 3  (0.281) |
|  | Policy publicity | 4  (0.295) | 5  (0.369) | 1  (0.074) | 0  (0) |  | 0  (0) | 6  (1.217) | 1  (0.203) | 0  (0) |  | 0  (0) | 8  (0.750) | 7  (0.656) | 1  (0.094) |
|  | Total | 75  (5.539) | 711  (52.511) | 265  (19.572) | 0  (0) |  | 0  (0) | 369  (74.848) | 19  (3.854) | 3  (0.609) |  | 0  (0) | 638  (59.794) | 140  (13.121) | 7  (0.656) |
| Demand-based | Government purchasing | 3  (0.222) | 6  (0.443) | 1  (0.074) | 0  (0) |  | 0  (0) | 2  (0.406) | 0  (0) | 0  (0) |  | 0  (0) | 3  (0.281) | 1  (0.094) | 0  (0) |
|  | Policy subsidies | 1  (0.074) | 8  (0.591) | 6  (0.443) | 0  (0) |  | 0  (0) | 5  (1.014) | 1  (0.203) | 0  (0) |  | 0  (0) | 13  (1.218) | 1  (0.094) | 0  (0) |
|  | Service outsourcing | 3  (0.222) | 29  (2.142) | 11  (0.812) | 0  (0) |  | 0  (0) | 19  (3.854) | 0  (0) | 0  (0) |  | 0  (0) | 21  (1.968) | 3  (0.281) | 1  (0.094) |
|  | Market cultivation | 2  (0.148) | 16  (1.182) | 0  (0) | 0  (0) |  | 0  (0) | 5  (1.014) | 0  (0) | 0  (0) |  | 0  (0) | 14  (1.312) | 1  (0.094) | 0  (0) |
|  | Demonstration pilots | 8  (0.591) | 34  (2.511) | 11  (0.812) | 0  (0) |  | 0  (0) | 17  (3.448) | 2  (0.406) | 0  (0) |  | 0  (0) | 39  (3.655) | 12  (1.125) | 2  (0.187) |
|  | Total | 17  (1.256) | 93  (6.869) | 29  (2.142) | 0  (0) |  | 0  (0) | 48  (9.736) | 3  (0.609) | 0  (0) |  | 0  (0) | 90  (8.435) | 18  (1.687) | 3  (0.281) |
| Total | | 104  (7.681) | 934  (68.981) | 316  (23.338) | 0  (0) |  | 0  (0) | 468  (94.929) | 22  (4.462) | 3  (0.609) |  | 0  (0) | 869  (81.433) | 188  (17.619) | 10  (0.937) |
